# Network-Slice-AwareVNF Orchestration for Mission-Critical IoT in 6G Networks: A Multi-Objective Deep Reinforcement Learning Approach

**DOI:** 10.3390/s26196023

**Published:** 2026-09-23

**Authors:** Abdulrahman K. Alnaim, Ahmed M. Alwakeel

**Affiliations:** 1 Department of Management Information Systems, College of Business Administration, King Faisal University, Alahsa 31982, Saudi Arabia; 2 Faculty of Computers & Information Technology, University of Tabuk, Tabuk 71491, Saudi Arabia; aalwakeel@ut.edu.sa

**Keywords:** 6G networks, cloud computing, mission-critical IoT, multi-objective deep reinforcement learning, network functions virtualization, network slicing, Pareto optimization, predictive scaling, temporal convolutional network, VNF orchestration

## Abstract

Mission-critical Internet of Things (MC-IoT) applications, such as remote surgery, autonomous drone swarms, industrial safety monitoring, etc., have ultra-stringent requirements for reliability (99.999%), latency (sub-millisecond) and inter-slice isolation that require dedicated network slices with carefully orchestrated virtual network function (VNF) chains. While prior solutions to VNF orchestration seek to merge often conflicting requirements into a single objective with a scalar reward, or to statically configure the allocation of resources that fail to adjust to dynamic mission-critical workloads, both strategies are inadequate. In this paper, we propose a network slicing-aware NFV orchestration framework called SliceNFV, which uses multi-objective deep reinforcement learning (MO-DRL) to optimize the resource allocation of VNFs per slice, the inter-slice isolation enforcement, and the efficiency of the cross-slice resource sharing. It is formulated as the orchestration problem as a multi-objective constrained Markov decision process (MO-CMDP), and a novel Pareto-Conditioned Proximal Policy Optimization (PC-PPO) algorithm is developed to learn a portfolio of non-dominated orchestration policies, allowing the operator to choose operating points along the reliability–efficiency frontier without retraining. A temporal convolutional network (TCN) is a scalable network that uses a predictive slice-aware VNF scaling mechanism to forecast the arrival of mission-critical events and scale resources in advance to avoid SLA breaches. The results of extensive simulation-based assessment across three progressive scenarios—steady-state operation, mission-critical demand surge and infrastructure degradation—highlight that SliceNFV offers 99.998% reliability when operating under an SLA violation rate of 0.08%, whereas all other baselines exceed their corresponding SLAs; in addition, SliceNFV significantly improves Pareto hypervolume by 36.4% over traditional multi-objective approaches while increasing infrastructure utilization to 76%.

## 1. Introduction

The sixth-generation (6G) wireless network is envisioned as an intelligent and highly programmable network fabric capable of supporting diverse services such as extended reality, autonomous industrial networks, public safety systems, and massive Internet of Things (IoT) deployments [1,2]. Among these services, mission-critical IoT (MC-IoT) applications require particularly strict performance guarantees, including ultra-low latency, very high reliability, and strong service isolation. Examples include remote surgical systems, autonomous drone swarms, cooperative vehicle platoons, and industrial safety monitoring [2,3].

Unlike conventional IoT services, MC-IoT applications may involve severe consequences when latency, reliability, or isolation requirements are not satisfied. A communication failure in remote surgery, industrial safety monitoring, or emergency drone coordination can lead to safety risks, infrastructure damage, or cascading operational disruption [4]. Therefore, supporting MC-IoT in 6G requires more than best-effort resource allocation; it requires adaptive and service-aware orchestration capable of balancing multiple conflicting quality-of-service (QoS) objectives.

Network slicing has been identified as the cornerstone approach to 5G and beyond to provide support for service diversity from shared physical infrastructure [5]. The segmentation of the network into end-to-end virtual networks allows the allocation of dedicated resources, protocol configuration, and management policies for each slice, based on the service that the slice is supposed to support [6]. The 3GPP standardization process has steadily evolved the slicing framework in Releases 15–18, covering the definition of mechanisms for the creation, selection and lifecycle management of slices [7]. However, with the advent of 6G, the slicing challenge becomes much more complex: the massive proliferation of IoT populations, the wide variety of services across MC-IoT verticals, and the need for real-time adaptive orchestration all demand intelligence beyond that provided by static or rule-based slice management [8].

Network slicing is implemented with the help of Network Functions Virtualization (NFV), which is the technological foundation. This decoupling of network functions from proprietary network hardware appliances and the ability to deploy and scale them as Virtual Network Functions (VNFs) running on top of general-purpose infrastructure allows the on-demand deployment of created network services and the ability to chain them together [9]. In this paradigm, every network slice is realized as a Service Function Chain (SFC) with ordered VNFs, all of which realize the required service logic. The VNF orchestration problem, which is NP-hard in its general form [10], is the challenge of optimally placing, configuring and dynamically scaling these VNFs on a distributed, multi-tier NFV infrastructure, while at the same time enforcing per-slice QoS constraints and maintaining inter-slice isolation. However, in 6G-MC-IoT applications, the problem also has other facets: resources allocated to one slice need to be balanced with the other slices having conflicting applications and goals (e.g., giving excess resources to one critical slice increases reliability, but degrades the reliability of another slice), decisions need to be made at millisecond time scales under stochastic traffic dynamics, and the infrastructure itself is heterogeneous, spanning different edge, fog and cloud tiers with different computational profiles.

Traditional optimization methods, including integer linear programming, heuristics, and meta-heuristics, can be effective in static or slowly changing environments but are less suitable for real-time orchestration in dynamic 6G-IoT deployments [10]. Deep reinforcement learning (DRL) has therefore become a promising alternative because it can learn adaptive resource-management policies through interaction with the environment [11,12,13]. However, many existing DRL-based slicing approaches rely on single-objective formulations or fixed scalar rewards. Such formulations require the operator to define one trade-off in advance and do not explicitly explore the Pareto-optimal balance among latency, reliability, isolation, and resource efficiency [14].

Multi-objective reinforcement learning (MORL) addresses this limitation by learning policies that represent different non-dominated trade-offs among competing objectives [15]. Recent advances such as preference-conditioned policies and Pareto-based learning have shown strong potential in complex decision-making domains. Nevertheless, their use for network-slice-aware NFV orchestration in mission-critical 6G IoT remains limited, particularly when predictive VNF scaling and inter-slice isolation are considered together.

Motivated by this gap, this paper proposes SliceNFV, a network-slice-aware NFV orchestration framework for mission-critical IoT services in 6G networks. SliceNFV formulates the orchestration problem as a multi-objective constrained Markov decision process (MO-CMDP) and introduces a Pareto-Conditioned Proximal Policy Optimization (PC-PPO) algorithm to learn a set of non-dominated orchestration policies. In addition, a temporal convolutional network (TCN)-based predictive scaling mechanism is integrated to forecast mission-critical demand changes and initiate VNF scaling before SLA violations occur.

The main features of this paper are recapped below:

(C1) We introduce a new comprehensive network slicing-aware NFV (6G) orchestration framework, SliceNFV, which consists of a hierarchical slice management layer and an intelligent MO-DRL orchestration engine, to optimize VNF lifecycle operations from end-to-end (e2e) across heterogeneous edge–fog–cloud infrastructure tiers in mission-critical IoT.

(C2) We model the problem of joint VNF resource allocation, inter-slice isolation and cross-slice efficiency optimization as a multi-objective constrained Markov decision process (MO-CMDP), and carefully define the state space, action space, multi-dimensional reward structure and constraint set to reflect the unique characteristics of mission-critical IoT service chains in 6G network slices.

(C3) We present Pareto-Conditioned Proximal Policy Optimization (PC-PPO), a multi-objective deep reinforcement learning algorithm using a preference-conditioned policy network which allows us to efficiently learn a variety of non-dominated orchestration policies, putting a network operator in the position to choose operating points along the reliability–efficiency trade-off, according to the current operational priorities.

(C4) We design a predictive slice-aware VNF scaling mechanism that leverages a temporal convolutional network (TCN) to predict patterns of mission-critical events and proactively initiate VNF scaling actions, thus minimizing reactive SLA violations by making adjustments to resource provisioning before the time of peak demand.

(C5) We evaluate SliceNFV under representative 6G-IoT slicing scenarios, including remote surgery, autonomous drone swarm, and industrial safety monitoring. The simulation results show that SliceNFV achieves high reliability, reduces SLA violations, and improves infrastructure utilization compared with representative static, heuristic, single-objective DRL, and weighted-sum MORL baselines under the evaluated assumptions.

The rest of this paper is organized as follows. The related literature of network slicing, NFV orchestration, and deep reinforcement learning for resource management in 5G/6G networks is reviewed in Section 2. In Section 3, the system model and problem formulation are introduced, comprising network architecture, slice taxonomy and the problem formulation of MO-CMDP. SliceNFV Methodology is presented in Section 4, which includes the design of the PC-PPO algorithm and the predictive VNF scaling mechanism. The experimental setup, simulation environment and the evaluation metrics are described in Section 5. The findings from the various sections are presented in Section 6 and discussed in detail with comparison. Finally, Section 7 concludes the paper, outlines the limitations of the present study and proposes directions for future research.

## 2. Related Work

In view of the proposed SliceNFV framework, this section analyzes, through four thematic areas, the existing literature that is most relevant to the proposed framework: network slicing for 5G/6G IoT services, NFV orchestration and VNF placement, deep reinforcement learning for network resource management, and multi-objective optimization in network slicing. In each of these areas, we outline the main contributions, methodological strengths and weaknesses, and open research questions that collectively inspire the current project.

### 2.1. Network Slicing for 5G/6G IoT Services

Network slicing has advanced quickly from a concept to a key feature of 5G system design, and is now being scaled out to 6G with much more ambitious goals. One of the first comprehensive works on network slicing-based 5G systems was presented by Zhang et al. [16], who presented a logical network architecture to handle mobility management between the access networks as well as joint power and subchannel allocation under inter-slice resource sharing. Their formulation set the design principle in place for all subsequent slicing work—ensure each slice has its own management system, but share the same physical substrate. The model of resource allocation for spectrum sharing in [16], however, was limited to two-level sharing and did not account for the multi-objective framework that is needed when both slices have conflicting requirements such as URLLC and massive machine-type communication (mMTC) for the same infrastructure.

Beyond 5G and 6G slicing have presented new requirements that the original slicing paradigm could not have foreseen. In [17], Rafique et al. introduced a comprehensive survey of beyond-5G network slicing for smart city applications, proposing a sliced end-to-end architecture, which covered radio access network (RAN), transport and core domains. They discussed scalable and adaptive slicing as a key technology for addressing smart city needs, as well as the challenges of inter-slice isolation and multi-domain management, and various AI/ML-based orchestration techniques. This work offered an architectural roadmap but did not provide any particular optimization algorithms required to implement the proposed vision, which translates the architectural concepts to operational intelligence, is left as an open problem.

Phyu et al. [18] gave a comprehensive review of machine learning techniques throughout the entire lifecycle of network slicing, breaking down the machine learning methods for slice admission, resource allocation and assurance. They found that supervised and unsupervised learning techniques have been extensively used for traffic prediction and anomaly detection in sliced networks, while reinforcement learning techniques appear to be most suitable for dynamic resource management tasks that require long-term optimization and are faced with a non-stationary environment. The survey pointed out, though, that the current RL-based slicing solutions are mostly only applicable to single-objective formulations and lack the capability to concurrently balance multiple competing slice KPIs.

Recently, Ebrahimi et al. [19] took a broad overview of the resource management problem throughout the entire range of 5G to 6G network slicing, covering the evolution from single-domain slicing to end-to-end cross-domain slicing. Their critical analysis highlighted that the majority of resource management solutions fit into one of the three domains—RAN, transport, core—and do not take into consideration the interdependencies among them, which are necessary to provide a true end-to-end slice optimization solution. The vision of the survey is for integrated approaches that are driven and aided by artificial intelligence (AI) and are capable of collectively managing the resources across all domains, while maintaining per-slice SLA constraints, which our proposed framework directly enhances.

### 2.2. NFV Orchestration and VNF Placement

The VNF placement problem, which involves determining the locations to place the instantiated VNFs in a distributed NFV infrastructure to optimize one or more performance metrics while respecting the capacity constraints, has been the focus of much research interest. Herrera and Botero [20] gave a thorough overview of the different resource allocation methods in NFV environments, which can be categorized as exact optimization (such as ILP), heuristic and meta-heuristic. Using exact methods, they found that they can deliver optimal solutions but have exponential computational complexity and, hence, are unsuitable when orchestration must be performed in real-time in large-scale networks; heuristics, on the other hand, have low complexity and are suitable for large-scale networks, as well as for use in other network topologies, but may not always deliver optimal solutions or offer any guarantees of their performance.

The challenge is much greater when VNFs are to be chained together with specific orderings and latency requirements, also known as Service Function Chains (SFCs). Bari et al. [21] transformed the VNF placement and the SFC embedding problem into an ILP to minimize the operational cost, and came up with heuristic algorithms for scalable solutions. Throughput optimization and delay guarantee (TO-DG) is their framework, which highlighted the need to consider fundamental overheads in the resources and the shareability of VNF instances, which many other formulations had ignored. However, the single-objective cost minimization formulation used in [21] does not reflect the multi-dimensional trade-offs between latency, reliability and resource efficiency that are typical in mission-critical IoT slicing use cases.

Following the tradition of delay-guaranteed SFC placement in multi-domain IoT networks, Liu et al. [22] proposed a multi-objective optimization model for placing SFCs in multi-domain IoT networks, which aims to maximize service acceptance ratio and minimize operation cost while meeting delay constraints. They have proposed parallelization of VNFs to lower the latency of SFCs, which is applicable to latency-sensitive IoT services especially. This work makes a significant contribution to an understanding of multi-domain SFC placement, but employs traditional optimization algorithms, which are not suitable for adapting to the dynamic traffic pattern of mission-critical environments in the context of IoT.

### 2.3. Deep Reinforcement Learning for Network Resource Management

Since 2020, the application of deep reinforcement learning to network slicing resource management has picked up significantly due to the urgent need for intelligent and adaptive orchestration that can adapt to the dynamic network environment without the need for rules or traffic models to be written manually. Hurtado Sánchez et al. [23] conducted a systematic survey on approaches for network slicing resource management based on DRL, categorizing them based on the DRL algorithm used (DQN, DDPG, PPO, SAC), the function of resource management (allocation, scaling, migration), and the evaluation methodology (simulation, real-world implementation). They also found that PPO-based methods are better suited for network slicing applications, owing to their stable training dynamics and their capacity to work with continuous action spaces, but most of the existing literature considers only simplified applications which do not fully reflect the complexity of multi-slice, multi-tier deployments in 6G.

In the specific application of DRL, Cai et al. [12] introduced a framework for online resource allocation in network slicing which learns a set of allocation policies through direct experiences in a simulated slicing environment. They formulated the resource allocation problem as a constrained MDP and used the PPO agent to maximize the long term network utility under the constraint of per-slice resources. The experimental results showed that the framework outperformed static allocation and greedy baselines, but the reward formulation based on a single objective did not allow for exploring trade-offs between competing slice objectives.

Rezazadeh et al. [13] considered federated DRL (FDRL) agents to jointly learn the slicing policies while avoiding sharing raw network data, which was a state-of-the-art advancement. They have developed a specialization process, which enabled various agents to specialize in various types of slices, thus enhancing the convergence speed and end policy quality. This federated architecture solved the scalability issue that was present in centralized DRL strategies, but did not introduce multi-objective optimization, instead using a scalar reward consisting of a combination of latency and throughput metrics weighted by pre-defined values.

In the multi-access edge computing (MEC) network slicing for utility optimization, Wang et al. [24] proposed a TD3-based twin-actor approach. They used a dual-actor architecture to mitigate the overestimation bias typical of actor–critic approaches and proved enhanced stability of resource allocation. This work’s MEC theme is especially pertinent to mission-critical IoT, where processing at the edge is required to satisfy the tight latency constraints. The single-objective utility function used, however, hides the individual per-slice performance guarantees mission-critical operators need to know about.

In [25], Sulaiman et al. introduced a multi-agent DRL method for the joint problem of network slicing admission control and resource allocation, showing that multi-agent formulations are superior in terms of convergence time and infrastructure provider’s revenues. Their research gave significant contribution to the concept of decomposing the slicing problem among several mutually collaborating agents. However, the goal of maximizing revenues does not directly consider reliability or isolation measures that are most dominant in mission-critical environments.

### 2.4. Multi-Objective Optimization in Network Slicing

Due to the fact that network slicing is in itself a multi-objective concept, there has been a growing number of studies on multi-objective solutions for slice management. Wang et al. [14] gave a thorough introduction to machine learning as an essential technology for Pareto-optimal wireless networks, summarizing the use of multi-objective optimization in different generations of wireless networks. In problems where the surface of the trade-off is not convex, or where the operators want to obtain the entire set of possible operating points, their analysis has shown that Pareto-based approaches are theoretically better than scalarization, which is typical of the multi-slice orchestration problem.

In the particular case of MORL, Yang et al. [15] introduced a generalized algorithm for multi-objective reinforcement learning which learns a single policy based on a preference vector and allows to select different trade-off points after training without retraining the policy. This envelope Q-learning method was shown to be able to efficiently approximate a complex Pareto Front in multi-objective environments where there are conflicting objectives. On this basis, Cai et al. [26] further developed the notion of Pareto-optimality to distributional Pareto-optimality, which not only involves the expected values of the various objectives but also the return distributions over the objectives. They developed their distributional Pareto-optimal MORL (DPMORL) framework that was able to meet multiple distributional preferences, which is especially important for mission-critical applications for which tail-risk performance (e.g., worst-case latency) is more important than average performance.

Although there are significant developments in MORL theory, limited efforts have been put into applying multi-objective optimization to network slicing. In their survey on machine learning-driven network slicing throughout the entire life cycle of a slice, Donatti et al. [27] noted that, although machine learning has been widely used for traffic prediction and anomaly detection, the use of multi-objective machine learning in slice orchestration is still in its infancy. The currently available multi-objective slicing algorithms mostly use evolutionary-based approaches like NSGA-II or weighted Tchebycheff decomposition, which necessitate full re-optimization when preferences change and are not able to offer the policy adaptation required by real-time mission-critical orchestration.

### 2.5. Summary and Research Gap

The key capability requirements for mission-critical NFV orchestration in 6G networks are compared with the works reviewed in Table 1. Seven dimensions are assessed: Network Slicing Management, NFV/VNF Orchestration, decision-making for DRL, Multi-Objective Optimization, Mission-Critical IoT Requirements, 6G-specific considerations and predictive scaling mechanisms.

From this comparison, some important points may be drawn as follows: First, though network slicing architectures and NFV orchestration mechanisms have advanced to a significant degree, they have developed independently, with none of the works reviewed having jointly optimized the management of QoS in network slices and orchestration of the VNF lifecycle in a single architecture. Second, DRL-based approaches have been shown to achieve good performance over traditional optimization approaches in the context of dynamic resource management, but most of the DRL approaches take the mission-critical KPIs as a scalar reward, or they ignore their multi-dimensional nature. Third, recent advances in MORL have enabled the generation of highly effective algorithms for generating Pareto sets of policies, which have yet to be used in the network slicing–NFV orchestration domain, not to mention the particular requirements for reliability and isolation of mission-critical IoT in 6G. Fourth, none of the existing work includes a predictive VNF scaling feature that predicts the timing of arrivals of mission-critical events, which is needed for guaranteeing SLA compliance when serving bursty MC-IoT traffic.

To overcome these limitations, the proposed SliceNFV framework includes (i) network slicing management and NFV orchestration in the same framework, (ii) the formulation of the orchestration problem as a multi-objective constrained MDP, where the key performance indicators (KPIs) of mission-critical slices are explicitly modeled, (iii) the development of a Pareto-Conditioned PPO algorithm that enables the discovery of a set of non-dominated policies without needing to specify the preferences a priori, and (iv) the incorporation of a predictive VNF scaling mechanism designed to the temporal dynamics of mission-critical event arrivals. As can be seen from the last row in Table 1, SliceNFV is the first framework to combine all the 7 capabilities to provide service orchestration for the 6G-MC-IoT services.

## 3. System Model and Problem Formulation

This section introduces the system model used for the SliceNFV framework and formalizes the multi-objective VNF orchestration problem for mission-critical IoT in 6G. The general architecture of the network is explained in great detail, first with the three-tier infrastructure topology and communication model. We then describe the slice taxonomy, the NFV Service Function Chain model and, finally, formulate the orchestration problem as a multi-objective constrained Markov decision process (MO-CMDP). The overall architecture of the system model is shown in Figure 1.

### 3.1. Three-Tier Infrastructure Topology

We view a 6G network that is deployed in a geographically extended metropolitan area supporting a diverse user base of mission-critical IoT devices. The NFV infrastructure consists of a three-tier hierarchical topology: edge tier, fog tier, and cloud Tier, all of which are the computational substrate for NFV hosting, as shown in Figure 1. The multi-tier architecture corresponds to the edge–fog–cloud continuum that has become the reference design for the latency-capacity trade-off needed in latency-sensitive 6G deployments [28] and enables VNFs to be deployed at the infrastructure tier best suited to the latency-capacity requirement of each slice.

Edge Tier. The edge tier comprises four edge nodes (n_7_, n_8_, n_9_, n_10_, as shown in Figure 1), which are co-located with one of 6G’s next-generation base stations (gNB). These nodes provide access latency < 1 ms (one-way propagation delay) and are limited only in terms of computation power at 8 GHz CPU capacity, 16 GB of RAM, and 1 GB of fronthaul bandwidth per node. The remote surgery slice, which requires end-to-end latency to be kept less than 1 mS, is an example of the most latency-sensitive slice, and VNFs are preferred to be placed at the edge tier. Edge nodes are situated near IoT devices and are the first point of contact for all uplink traffic, where initial processing can be executed, including encoding, compression, and protocol translation before the data is transferred to higher layers if needed.

Fog Tier. The fog tier consists of three fog nodes, denoted as $N_{f}=\left\{n_{4},n_{5},n_{6}\right\}$, deployed at network aggregation points such as metro-area central offices or regional data hubs. The fog nodes have moderate computational power (24–32 GHz CPU, 48–64 GB RAM) and intermediate access latency (1–5 ms). Edge nodes are connected to fog nodes through aggregation links, which can achieve 5 Gbps. The fog tier is mainly used to host more demanding VNFs than those that can be deployed at the edge and still be latency-sensitive, but can still be latency-tolerant when deployed in the cloud, and also to serve as an aggregation point where data streams from a number of edge nodes are aggregated and then processed in the cloud. For instance, the autonomous drone swarm slice can be constructed with moderate computational requirements for the coordination VNFs that can be deployed at the fog tier to avoid the latency from the cloud tier, while the trajectory optimization can be realized in real-time.

Cloud Tier. The cloud tier consists of a centralized data center with high compute resources (64–128 GHz CPU, 256–512 GB RAM) and 10 Gbps backbone links and is composed of cloud servers (n_1_, n_2_, n_3_) of Nc. Access latency of the cloud tier is from 5 to 20 ms based on geographic distance and routing path. Although it has a longer latency, the cloud tier is essential for those VNFs that require high computational processing, such as deep analytics, long-term data logging, and AI model inference, which are not possible within the edge and fog nodes’ capabilities. For example, the industrial safety monitoring slice places all its heavy analytics and logging VNFs in the cloud tier and all its light data collection and alarm VNFs nearer to the edge.

### 3.2. Communication Model

The infrastructure is formally represented as a weighted directed graph G = (N, L) with the complete set of |N| = 10 infrastructure nodes N = Ne ∪ Nf ∪ Nc and the set of communication links L. The resource capacity vector cn is a characterization of each node n ∈ N, representing its CPU (GHz), memory (GB), and bandwidth (Gbps).

Each communication link l ∈ L has its bandwidth bl and propagation delay dl. The intra-edge links are at 1 Gbps with sub-millisecond delay, while the edge-to-fog aggregation links are at 5 Gbps and have a delay of 1–5 ms; the fog-to-cloud backbone links are at 10 Gbps with a delay of 5–20 ms; and the intra-cloud links are at 100 Gbps with negligible delay. The centralized SliceNFV orchestrator is modeled as a logically centralized global control-plane entity, separate from the cloud, fog, and edge resource tiers. It maintains a global view of node resources and link occupancy across all tiers and makes VNF lifecycle decisions at time steps t=1,2,…,T, where each step represents a decision epoch of length Δt [29]. The infrastructure node specifications used in the simulation are summarized in Table 2.

### 3.3. Slice Taxonomy for Mission-Critical IoT

The network consists of K network slices (k = 1, 2, …, K). Each slice is assigned to a specific type of service class for MC-IoT having different Quality of Service Requirements. Based on the definition of URLLC slices in 3GPP and the recent mission-critical IoT literature [4,30,31,32], we have identified three representative slice types. In the simulated environment, these three slice types are assumed to coexist over the same shared edge–fog–cloud NFV infrastructure, representing heterogeneous mission-critical services operated concurrently by the same infrastructure provider.

Slice Type 1—Remote Surgery (RS): It is meant to be used in real-time haptic feedback and high-definition video streaming in telesurgical systems. It requires the highest latency and reliability requirements of all slice types, which are less than 1 ms end-to-end and 99.999% reliability. The above SFC F_1_ is composed of five VNFs: Encode, Compress, Encrypt, Forward, Decrypt. As these VNFs are highly latency-sensitive, most of them are then deployed at the edge tier, while the Decrypt function may be offloaded to the fog nodes if the edge tier is overloaded.

Slice Type 2—Autonomous Drone Swarm (ADS): This slice is for cooperative autonomous drone formations that undertake search and rescue or infrastructure inspection tasks. It should be low latency (less than 5 milliseconds) for inter-drone coordination messages, and it should be high reliability (99.99%). The SFC F_2_ is composed of four VNFs: Aggregate, Process, Route, and Coordinate. The Route and Coordinate VNFs are usually deployed at fog nodes, whereas the Aggregate and Process VNFs are deployed at edge nodes.

Slice Type 3—Industrial Safety Monitoring (ISM): The slice is used for linking distributed sensor networks in a hazardous industrial environment. It requires moderate latency (<10 ms), high reliability (99.99%) and the ability to process periodic bulk sensor data uploads and sporadic alarm events. The SFC F_3_ consists of five VNFs: Collect, Filter, Analyze, Alert, and Log. The Collect and Filter VNFs are deployed in the edge or fog tier and the Analyze and Log VNFs are deployed in the cloud tier. The Alert VNF sits in the fog tier to achieve a compromise between responsiveness and analytical depth. The QoS requirements and SFC characteristics of the three mission-critical IoT slice types are summarized in Table 3.

### 3.4. VNF and Service Function Chain Model

A network slice k is instantiated as a Service Function Chain (SFC) made up of an ordered sequence of Virtual Network Functions (VNFs) {fk,1, fk,2, …, fk,Mk} with Mk number of VNFs in the chain. Each VNF fk,m is represented by a vector of computational resource demands that indicate the CPU and memory needs [20]. A binary variable $x_{k},m,n(t)\in \{0,1\}$ is used to represent the VNF placement decision.

Following standard SFC/VNF modeling principles for latency and reliability evaluation in NFV environments [20,21], we define the slice-level end-to-end latency, reliability, and isolation index for the proposed SliceNFV system as follows. Equations (1)–(3) are adapted to the proposed multi-slice edge–fog–cloud setting.

*D_k_*(*t*) = ∑*_m_ d_proc_*(*f_k_*_,*m*_, *n*) + ∑*_m_ d_link_*(*l_m_*_,*m*+1_) (1)

*R_k_*(*t*) = ∏*_m_ r_n_*(*n_m_*)⋯∏*_m_ r*_l_(*l_m_*_,*m*+1_) (2)

(3) $$I_{k\left(t\right)}=1-\frac{\sum_{n}\;\sum_{j}\neq_{k}\;\delta_{n,k,j}\left(t\right)}{\sum_{n}\;x_{k,n}(t)}$$

where $\delta_{n,k,j}\left(t\right)$ is a binary indicator for resource contention between slices *k* and *j* on node n. An isolation index of 1 indicates perfect isolation; lower values indicate increasing contention.

### 3.5. Multi-Objective Constrained MDP Formulation

Following standard constrained and multi-objective reinforcement learning formulations [15,33], we represent the VNF orchestration problem as a Multi-Objective Constrained Markov Decision Process (MO-CMDP), defined by $\left(S,A,P,R,C,\gamma \right)$, where S is the state space, A is the action space, P is the state transition function, R is the multi-dimensional reward function, C is the constraint set, and $\gamma \in \left[0,1\right)$ is the discount factor.

State Space. The orchestrator sees a composite state, which includes: (i) infrastructure state, residual resource vectors for all nodes; (ii) slice state, current placements of VNFs, measured latency, reliability, isolation, and traffic load of each slice; and (iii) temporal context, predicted mission-critical event indicators from the TCN module.

*s*(*t*) = *s_inf_*(*t*), *s_slice_*(*t*), *s_ctx_*(*t*) (4)

Action Space. The action simultaneously decides VNF placement and scaling decisions and continuous resource allocation quantities:

*a*(*t*) = *a_d_*(*t*), *a_c_*(*t*) (5)

Multi-Dimensional Reward. The MO-CMDP defines a vector-valued reward R(s, a) = (R_1_, R_2_, R_3_):

(6) $$\;R_{1\left(t\right)}=\sum_{k}w_{k}\cdot \left.\left[\alpha_{1}(1-\frac{D_{k}\left(t\right)}{d_{k}max})+\alpha_{2}(R_{k}\left(t\right)-r_{k}min)\right.\right]$$

(7) $$\;R_{2\left(t\right)}=\frac{1}{K}\sum_{k}I_{k\left(t\right)}$$

(8) $$\;R_{3\left(t\right)}=\frac{1}{N}\sum_{n}\left.\left[1-{\left|u_{n\left(t\right)}-u^{*}\right|}^{2}\right.\right]$$

Constraint Set. Four constraints are enforced: (C1) node capacity; (C2) placement uniqueness; (C3) SLA compliance; and (C4) minimum isolation thresholds.

### 3.6. Pareto-Optimal Objective

Based on the standard Pareto-dominance concept used in multi-objective optimization and MORL [14,15], the goal is to find a set of Pareto-optimal policies $\Pi^{*}$ such that no feasible policy dominates another policy in the set.

*J_i_*(*π*′) ≥ *J_i_*(π*) ∀*i* ∈ {1,2,3} *and*
*J_j_*(*π*′) > *J_j_*(π*) *for some*
*j* (9)

The resulting Pareto set represents a frontier of non-dominated orchestration strategies for the operators during runtime without retraining. In Section 4, we will give an algorithm to determine this set.

## 4. Proposed SliceNFV Methodology

The full method for the SliceNFV framework is provided in this section. The four-layer framework architecture and its operation are detailed in Section 4.1. The Pareto-Conditioned PPO (PC-PPO) algorithm is described in Section 4.2 and it is based on the architecture of the neural network, training procedure and the Pareto Archive management. Section 4.3 describes the predictive slice-aware VNF scaling mechanism that is based on a temporal convolutional network (TCN). During live deployment, the trained framework, described in Section 4.4, will put these things into practice.

### 4.1. Framework Architecture

The SliceNFV framework is split into four hierarchical levels, as shown in Figure 2. Every layer has a clear responsibility and uses a common interface to communicate with the next layer down and up, where state observations are passed down and orchestration actions are passed up.

Layer 1—Mission-Critical IoT Device Layer. This is the lowest layer in which the IoT devices from the three MC-IoT verticals (remote surgery, drone swarm, and industrial safety) create uplink traffic. The devices generate different types of traffic: remote surgery devices send continuous high-fidelity streams of 50–200 Mbps, drone swarm units send bursty coordination messages with swarm maneuvers ranging from 10 Mbps to 500 Mbps and industrial sensors send periodic telemetry at 1–50 Mbps, and sporadic alarms during safety events as bursts at 10 Mbps to 500 Mbps. The first edge node to which traffic arrives in the system is the nearest one.

Layer 2—NFV Infrastructure Layer. This layer is used for running the VNF instances over the three-tier edge–fog–cloud topology described in Section 3.1, which consists of 10 infrastructure nodes: four edge nodes, three fog nodes, and three cloud servers. Typically, each node has a lightweight telemetry agent that sends back to the orchestrator the status of the node resources (CPU, memory, bandwidth) and the status of the VNF instance every Δt = 100 ms. The infrastructure layer also keeps track of inter-node links’ link-level characteristics such as propagation delay, packet loss rate, and available bandwidth.

Layer 3—Slice Management Layer. The layer helps ensure logical separation between the three concurrent network slices. It keeps track of the current state of the SFC (which VNFs are instantiated, where they are placed, and how they are chained), real-time QoS measurements (end-to-end latency D_k_(t), reliability R_k_(t), and isolation index I_k_(t) as defined in Equations (1)–(3)), and current traffic load λ_k_(t). Slice isolation policies are enforced through the slice management layer, which monitors resource contention events between slices that share the same physical nodes and reports resource contention violation events to the orchestrator.

Layer 4—MO-DRL Orchestration Engine. This is the part of the framework that is responsible for intelligent decision-making and would include four tightly integrated modules. The PC-PPO Agent gets the composite state vector s(t) from Layers 2 and 3 and supplements it with predictive signals from the TCN predictor, which it returns as orchestration actions such as VNF placement, resource allocation, and scaling decisions. The Preference Selector receives the actual specification of the network operator as a priority value at runtime (as a priority vector ω* over the three objectives) and then conditions the agent’s policy based on this priority. The TCN predictor uses the recent traffic history to predict the number of arrivals of mission-critical events and the expected demand level for each slice. The VNF Lifecycle Controller converts the abstract decisions made by the agent into real ETSI MANO-compatible VNF lifecycle operations: VNF instantiation, horizontal/vertical scaling, live migration between nodes and graceful termination.

At each decision step t, (i) the infrastructure and slice layers provide the state s(t); (ii) the TCN predictor produces the next epoch’s demands and event probability estimates (forecast); (iii) the PC-PPO agent observes the state and augmented state [s(t), forecast, ω*] and chooses an action a(t); (iv) the VNF Lifecycle Controller takes action a(t) in the infrastructure; (v) the environment moves to state s(t + 1), and a vector-valued reward R(t) = (R_1_, R_2_, R_3_) is emitted; and (vi) the tuple (s(t), a(t), R(t) = (R_1_, R_2_, R_3_), s(t + 1)) is stored for policy improvement during training.

### 4.2. Pareto-Conditioned PPO (PC-PPO) Algorithm

The main contribution of this work is the extension of standard Proximal Policy Optimization [34] for the multi-objective setting, the algorithm called PC-PPO. The main idea is to condition the policy and value networks with a preference vector ω that is sampled from the 2-simplex $\Delta 2\;=\;\{\omega \;\in R^{3}\;:\;\omega i\;\geq \;0,\;\sum_{i=1}^{3}\omega i\;=\;1\;\}$. This conditioning allows a single neural network to learn an entire family of policies (one policy per possible compromise on latency-reliability, isolation and resource efficiency) instead of training a separate agent for each of these points on the trade-off curve.

Neural Network Architecture. The PC-PPO agent has the following structure of the neural network. The input layer has 64 dimensions, which is the aggregation of the state vector s(t) (10 nodes × 3 resource metrics + 3 slices × 5 KPI metrics + 16 TCN context features = 61 dimensions) and the 3-dimensional preference vector ω. This input is processed by a shared encoder made with three fully connected layers—256, 256, 128 neurons, and ReLU activations and layer normalization processes. The output of the encoder is split into two heads:

(i) Policy Head: Further, there is a policy head, which can be subdivided into a discrete sub-head and a continuous sub-head. The sub-head generates a categorical probability distribution of VNF placement and scaling decisions (which node to place each VNF on, scale up/down/migrate). This continuous sub-head generates the mean and log-standard-deviation of a clipped Gaussian distribution on the quantity of resources allocated to a VNF instance (the amount of CPU and memory to be assigned to the instance). Both sub-heads comprise 2 fully connected layers (128, 64 neurons).

(ii) Value Head: This head gives three independent scalar outputs: V_1_(s, ω), V_2_(s, ω), and V_3_(s, ω) for each objective. A final linear layer is attached to each value output, but the two hidden layers (128, 64 neurons) are shared with the value head trunk. Value estimates for each objective are needed to determine per-objective advantages and this in turn allows the use of preference-conditioned scalarization, which is the distinguishing feature of PC-PPO from standard single-objective PPO. Table 4 shows the PC-PPO neural network architecture specifications, and PC-PPO training hyperparameters in Table 5.

Training Procedure. The training process is briefly summarized in Algorithm 1 and shown in Figure 3. Each time a rollout episode starts, a preference vector ω is uniformly randomly sampled from the simplex Δ^2^. The agent then plays in the simulated 6G-IoT slicing environment for 2048 time steps, obtaining a sequence of state–action–reward tuples for the 6G-IoT slicing environment under the sampled preference. After each rollout batch, the per-objective advantages are calculated using Generalized Advantage Estimation (GAE) [35] with γ = 0.99 and λ = 0.95, and then scalarized into a composite advantage using the sampled preference weights. The policy and value networks are trained for 10 epochs of minibatch stochastic gradient descent (SGD) with a learning rate of 3 × 10^−4^ and a minibatch size of 256. This policy is a standard PPO clipped surrogate model with the value loss coefficient c_1_ = 0.5 and entropy bonus coefficient c_2_ = 0.01 to promote exploration. The policy is a standard PPO clipped surrogate model with the value loss coefficient c_1_ = 0.5 and the entropy bonus coefficient c_2_ = 0.01.

The key difference between standard PPO and this approach is that the shared encoder is trained with variations of ω in different episodes to acquire feature representations that are applicable to the whole of the Pareto front. The agent is trained over more than 5000 iterations of training and sees thousands of different preference vectors, thus covering the trade-off space broadly. Training is performed offline and requires approximately 10 million environment steps, taking about 8 h on a single NVIDIA A100 GPU (NVIDIA Corporation, Santa Clara, CA, USA). This offline training cost does not affect online orchestration, where the trained policy is used only for forward-pass inference.

Pareto Archive Management. At every 50 iterations, the current policy is tested on a fixed set of 21 equally spaced preference vectors defined on the simplex. The following table shows the results of an agent’s policy for each preference over 10 episodes of evaluation. The following table shows what the agent earns for each preference over 10 episodes of evaluation, where the mean return vector is $J={(J}_{1,}J_{2},J_{3})$. If the return vector is not dominated by any other existing return vectors in the Pareto Archive Π*, then the policy snapshot and the associated preference are inserted into the Pareto Archive Π*, and elements of the Pareto Archive that are now dominated by the new return vector are deleted. The archive quality indicator is the hypervolume indicator [36], which measures the volume of the objective space dominated by the archive relative to a reference point, and is used to monitor the quality and diversity of the archive. Training is regarded as converged when improvement in hypervolume is less than 0.1% for 500 (or more) iterations. The archive will generally contain 15–25 policies that are non-dominated at convergence.
**Algorithm 1.** PC-PPO Training ProcedureInput: Environment E, hyperparameters (Table 5)Output: Pareto Policy Archive Π*Initialize policy πθ, value heads Vφ, archive Π* ← ∅for iteration = 1 to 5000 do     Sample preference ω~Uniform(Δ^2^)     Collect trajectory τ = {(s,a,R,s′)} for 2048 steps using πθ(·|s, ω)     for each objective i ∈ {1,2,3} do         Compute advantages A_i_(t) via GAE(γ = 0.99, λ = 0.95)     end for     Scalarize: Aω(t) = ω_1_A_1_(t) + ω_2_A_2_(t) + ω_3_A_3_(t)     for epoch = 1 to K = 10 do         Sample minibatch B of size 256 from τ         Compute PPO clipped loss with ε = 0.2 using Aω         Compute value loss = ∑_i_ ω_i_(V_i_ − R_i__target)^2^         Update θ, φ via Adam (lr = 3 × 10^−4^)     end for     if iteration mod 50 == 0 then         Evaluate πθ on 21 test preferences → {J_1_, J_2_, J_3_}         Add non-dominated policies to Π*; prune dominated         Compute hypervolume(Π*); check convergence     end ifend forreturn Π*

### 4.3. Predictive Slice-Aware VNF Scaling

The problem with only reactive VNF scaling is that the mission-critical service has degraded by the time it realizes that there is a degradation and takes action to scale up. In some applications like remote surgery, a latency spike of 100 ms during a scaling transition may be enough to endanger the patient. SliceNFV responds to this with a proactive scaling mechanism based on a temporal convolutional network (TCN) [37] predicting mission-critical events and demand spikes.

Therefore, the predictive module is intended not only to reduce reactive SLA violations, but also to compensate for the actuation delay of VNF lifecycle operations by triggering resource adjustments before the predicted demand peak.

It should be noted that the TCN is not a standalone orchestration policy; it serves as a forecasting module that provides predictive context to the PC-PPO agent, while the final VNF placement, scaling, and resource-allocation decisions are made by the PC-PPO policy.

TCN Architecture. The TCN was selected for three reasons: (i) parallelizable computation, which allows for faster inference that is essential for real-time operation at 100 ms decision epochs; (ii) gradient flow stability during training, without the presence of recurrent connections; and (iii) by virtue of the dilation factors, the receptive field is flexible to capture short-term burstiness (drone swarm maneuvers) and longer-term periodicity (industrial sensor reporting cycles). The TCN is composed of four residual blocks, with two dilated causal convolutional layers (with kernel size three and dilated factors [1, 2, 4, 8]). With this setup, the receptive field is 24 = 16 time steps or 1.6 s of traffic history at Δt = 100 ms. Batch normalization, ReLU activation and dropout (rate = 0.1) are implemented in each residual block. The input to the TCN is a window of the last W traffic load observations (λ_k_(t) for each slice k) and measurements of resource usage metrics.

Prediction Outputs. TCN provides two outputs for each slice *k*: (a) the predicted traffic demand ${\hat{\lambda }}_{k}(t+1)$ or the next decision epoch, and (b) the predicted probability ${\hat{p}}_{k}(t+1)$ of a mission-critical event (e.g., a surgical emergency escalation, a drone collision-avoidance maneuver, an industrial safety alarm). The pre-training was done on 72 h of simulated MC-IoT traffic data, with a mean squared error loss for demand prediction and a binary cross-entropy loss for the estimation of the probability of an event, resulting in an MAPE of 8.3% for demand prediction and an AUC of 0.94 for event estimation.

Preemptive Scaling Trigger. If the predicted probability ${\hat{p}}_{k}(t+1)$ is above a threshold τ_k_ (which is prepared for each slice as follows: τ_k_ = 0.7 for RS, τ_k_ = 0.75 for ADS, and τ_k_ = 0.8 for ISM, based on the relative criticality of the slices), the scaling mechanism calculates a preemptive resource augmentation in proportion to the expected demand surplus:

(10) $$\Delta \rho_{k}(t)\;=\;\beta \text{·}\max(0,\;{\hat{\lambda }}_{k}(t+1)-\lambda_{k}(t))\cdots \rho_{\mathrm{kbase}}$$

where β = 1.2 is a scaling sensitivity parameter, which offers a 20% safety margin above the predicted demand, and ρ_k__base is the current base resource allocation for slice k. This enhancement signal is added to the state vector’s temporal context s_ctx(t) that is passed to the PC-PPO agent. The mechanism keeps the agent flexible in making globally coordinated decisions, since it is not passed through the agent but embedded into its state, while it still harnesses the lookahead information. The agent may decide to take action according to the prediction, or to ignore or reject it, based on the state of the infrastructure and the operator’s preferences.

### 4.4. Inference and Deployment

In the live deployment, the trained PC-PPO agent runs without gradient updates (lower half of Figure 3). The following deployment process is followed. During a surgery, the network operator determines a preference vector ω* that has operational priorities, such as ω* = (0.6, 0.2, 0.2) for latency-reliability or ω* = (0.2, 0.3, 0.5) for resource efficiency during off-peak hours. The Preference Selector fetches the best policy in the Pareto Archive that is closest to the policy in terms of Euclidean distance in preference space. The selected policy is fed into the inference engine and produces VNF orchestration actions at every 100 ms decision epoch used in the simulation.

It should be noted that the sub-millisecond requirement in this study refers to the end-to-end latency target of mission-critical services, not to the orchestration-control decision interval. The present experiments evaluate SliceNFV under a 100 ms control decision epoch and do not include a separate micro-benchmark of PC-PPO inference latency. Since the trained PC-PPO policy is a feed-forward neural network with approximately 287 K parameters, online inference is lightweight compared with offline training; however, we do not claim a guaranteed <1 ms control-decision latency without hardware-specific benchmarking. Achieving sub-millisecond control-loop latency in practical deployments would require optimized inference, local edge execution, or hierarchical control.

The most important advantage of PC-PPO is that when switching between different operating points on the Pareto frontier, you only need to change the preference input vector; no retraining, fine-tuning or policy re-loading is needed because the preference-conditioned network can evaluate any ω* during inference time. This allows the network to respond in real time to mission conditions and dynamically change its optimization strategy within milliseconds, for example, if a mission emergency procedure is triggered or a drone swarm is entering a collision-risk zone. The TCN predictor is deployed concurrently with the policy and continuously updates the demand forecasting and event probability information to the augmented state at every decision epoch, thus ensuring that the policy can be scaled proactively during the whole operation.

## 5. Experimental Setup

This section introduces the experimental setup, the baseline methods, the metrics used to assess the performance of the SliceNFV framework and the test scenarios evaluated. The experiments are carried out in a simulated environment (no physical testbed). This design decision is not coincidental, since a simulation environment allows evaluation of the system under reproducible conditions, systematic variation in workload parameters, and the ability to compare all the baselines in identical traffic and failure scenarios, which are hard to ensure on physical deployments [38].

### 5.1. Simulation Environment

The simulation environment is realized as a custom discrete-event simulator implemented in Python (v3.10, Python Software Foundation, Wilmington, DE, USA) using the Gymnasium interface specification interface specification [39] to be compatible with common DRL training frameworks. To implement the DRL agents, we use PyTorch (v2.1; PyTorch Foundation, San Francisco, CA, USA) and the Stable-Baselines3 library that offers a stable base implementation of PPO, and we extend this implementation to include multi-objective preference conditioning. All experiments are performed on an NVIDIA A100 80 GB GPU, AMD EPYC 7763 64-core CPU, 256 GB RAM and Ubuntu 22.04 LTS OS (Canonical Ltd., London, UK).

The simulator is based on the three-tier NFV infrastructure described in Section 3.1 (4 edge nodes, 3 fog nodes, 3 cloud servers; 10 nodes total) and the same resource capacity and link attributes are used as detailed in Table 2. The simulator runs at every decision epoch (Δt = 100 ms), updating the status of all infrastructure nodes, calculating per-slice QoS metrics (latency, reliability and isolation) as a function of the current VNF placement and traffic load, and, finally, executing the orchestration actions chosen by the agent. In the present simulation, VNF lifecycle actions are executed at the decision-epoch level, and separate execution-time distributions for VNF instantiation, horizontal/vertical scaling, live migration, and termination are not explicitly modeled. This abstraction allows all orchestration methods to be compared under identical traffic, resource, and failure conditions. However, in practical NFV deployments, these operations may introduce different actuation delays. SliceNFV mitigates this issue through predictive scaling: the TCN module forecasts upcoming demand changes and allows the PC-PPO agent to initiate scaling or migration before the SLA boundary is reached. In practice, the prediction horizon should be configured to be longer than the expected actuation delay of the selected VNF lifecycle operation, especially for slower actions such as instantiation and live migration. The number of arrivals of traffic for each slice is determined by stochastic models described in Section 5.2. The failures of nodes and links are injected stochastically and follow empirical telecom equipment reliability data per component [40].

The simulation assumptions are designed to approximate practical edge–fog–cloud NFV orchestration conditions, including heterogeneous infrastructure resources, tier-specific communication delays, stochastic mission-critical traffic, and failure events. However, the evaluation remains simulation-based and does not fully capture all production deployment factors, such as SDN control-plane delays, telemetry collection latency, container runtime overhead, VNF boot time, hardware-specific acceleration effects, and operator-specific management policies. Therefore, the reported results should be interpreted as simulation-based evidence of feasibility under the stated assumptions rather than direct proof of production deployment performance.

The 10,000 decision epochs (1000 s of simulated network operation) are used for each simulation run. The results are obtained over 30 independent evaluation runs with different environment random seeds. We report the results as mean ± standard deviation and include 95% confidence intervals computed using Student’s t-distribution.

### 5.2. Traffic Models

Each MC-IoT slice has stochastic traffic that is generated using a unique stochastic model with calibrations that take into account the temporal attributes of the target application:

Remote Surgery (RS) Slice: The traffic comes as a continuous flow modeled with a Gaussian process whose mean rate is μ_1_ = 120 Mbps and whose standard deviation is σ_1_ = 20 Mbps, representing the steady video and haptic data flow of an active surgical session. Mission-critical events (surgical emergencies requiring higher bandwidth usage for extra monitoring channels) are Poisson distributed with a rate of λ_1__event = 0.02 events per second, and each event incurs a demand change of 2× that lasts for 5–15 s.

Autonomous Drone Swarm (ADS) Slice: Traffic is modeled as a Markov-modulated Poisson process (MMPP) with two states: “cruise” (mean rate 30 Mbps) and “maneuver” (mean rate 350 Mbps). Swarm transitions between cruise and maneuver with a probability of 0.05/epoch and back to cruise with a probability of 0.15/epoch, which accounts for the bursty coordination of drone swarms for avoidance maneuvers or formation changes.

Industrial Safety Monitoring (ISM) Slice: baseline traffic is periodic, and it has a deterministic speed of 10 Mbps (sensor reporting cycles). To minimize the chance of any problems, the safety alarm events are modeled as a Poisson process with a rate of λ_3__event = 0.01 events per second and a burst of 40 Mbps for a duration of 3–8 s, which activates the system’s high-resolution monitoring, video feeds, and emergency communication channels.

### 5.3. Baseline Methods

We present a comparison between SliceNFV and four baseline methods that represent the current state of practice and state of the art in the area of network slicing resource management:

Baseline 1—Static Slicing (SS): Slices are statically assigned at initialisation according to estimates of peak demand for each of the resources and remain fixed throughout the system lifecycle. Dedicated nodes are used to allocate CPU, memory and bandwidth to each slice, without any sharing across slices. This is a minimum level of operation and is only a lower limit on adaptability.

Baseline 2—Greedy Heuristic (GH): At each decision epoch, a greedy and first-fit algorithm installs the VNFs on the node with the lowest occupation level at the time that meets the VNF requirements. The rule for scaling is based on a threshold: If the latency of the slice is more than 80% of the maximum allowable latency, then the resources are increased by 25%; if the resource usage of the slice is below 30%, then the resources are decreased by 15%. This is a heuristic baseline that is a very common practice.

Baseline 3—Single-Objective PPO (SO-PPO): A standard PPO agent, which is trained with a scalar reward combining all three objectives with fixed weights (ω = (0.5, 0.25, 0.25)). The network architecture and the hyperparameters used for training are the same as for PC-PPO (Table 5 and Table 6), except that the preference input is not included and the value head outputs a single scalar. This baseline removes the benefit of multi-objective optimization and is used to compare results with a similar DRL algorithm with no Pareto conditioning.

Baseline 4—Weighted-Sum MORL (WS-MORL): A multi-objective approach that trains K=5 separate PPO agents, each with a different fixed weight vector $\omega \in \left\{\left(0.8,0.1,0.1\right),\left(0.1,0.8,0.1\right),\left(0.1,0.1,0.8\right),\left(0.5,0.25,0.25\right),\left(0.33,0.33,0.34\right)\right\}$.

During deployment, the operator selects the pre-trained agent whose weight vector is closest to the current operational preference. This baseline represents a conventional weighted-sum MORL strategy with a limited fixed preference set. Since the number and distribution of preference vectors can affect Pareto-front coverage and hypervolume, the comparison with PC-PPO should be interpreted as comparing preference-conditioned policy learning against a fixed-weight multi-policy baseline, rather than as an exhaustive comparison under all possible preference discretizations.

Component-level clarification: The proposed SliceNFV framework combines PC-PPO-based multi-objective decision-making with TCN-based predictive context. The TCN module alone is not evaluated as an independent baseline because it does not generate orchestration actions; it only forecasts future demand and event probabilities. The non-predictive DRL baselines, including SO-PPO and WS-MORL, help indicate the effect of learned orchestration without the full predictive SliceNFV mechanism. A dedicated numerical ablation of PC-PPO without TCN is identified as an important extension for future experimental work.

### 5.4. Evaluation Metrics

All methods are assessed based on 6 metrics that cover the various aspects of the mission-critical NFV orchestration features:

(M1) Average End-to-End Latency: Average end-to-end latency over all the slices during the evaluation period, defined as $D^{-}$ = (1/K) ∑_k_ $D_{k}^{-}$. Lower numbers mean better latency performance.

(M2) Reliability (Packet Delivery Ratio): The average (across slices) proportion of packets delivered within the latency bound for each slice. Reliabilities closer to 1.0 are more reliable.

(M3) SLA Violation Rate: The ratio of the number of epochs at which the SLA requirements (latency or reliability) are violated in at least one slice. For mission-critical applications, this is the most critical measure that could result in life-threatening consequences if violated.

ISII: (M4) Inter-Slice Isolation Index: Average isolation index over all slices during the evaluation period as given by Equation (3). A higher score of 1.0 means that there is a greater degree of isolation.

Resource Utilization (M5): The average resource utilization of all the infrastructure nodes is the mean ratio of allocated to available CPU and memory resources. The range of 0.6 to 0.8 is recommended to be the optimum, giving a balance between efficiency and headroom for demand spikes.

(M6) Pareto Hypervolume: This metric is used to evaluate the quality and diversity of the trade-off front obtained by the multi-objective methods. In this study, the reported hypervolume improvement is calculated in a three-dimensional normalized objective space $J=\left(J_{1},J_{2},J_{3}\right)$, corresponding to SLA-oriented latency/reliability performance, inter-slice isolation, and resource-efficiency performance. The objective values are normalized to $\left[0,1\right]$, and the hypervolume is computed using the reference point $\left(0,0,0\right)$. This metric is reported only for SliceNFV and WS-MORL.

### 5.5. Evaluation Scenarios

All methods are assessed in three scenarios with increasing difficulty to challenge various facets of the orchestration framework. In all scenarios, the RS, ADS, and ISM slices run concurrently over the same physical infrastructure; therefore, the evaluation captures inter-slice resource sharing and isolation among heterogeneous mission-critical services.

Scenario 1—Steady-State Operation: All three slices are given nominal traffic conditions for 10,000 epochs of duration. Their rates of occurrence for mission-critical events are the base rate (Table 7). No failures are introduced to the infrastructure. This scenario is an attempt to measure how well the orchestra is organized at a baseline level in stable conditions and to set a baseline level of performance for all methods.

Scenario 2—Mission-Critical Surge: An active maneuver (similar to an active search-and-rescue scenario) is performed on the ADS slice while the RS slice has a 3× demand spike during epochs 3000 and 5000. The ISM slice resumes its normal functioning. This scenario tests the framework to withstand multiple peaks of demand on various critical slices and achieve SLA compliance.

Scenario 3—Infrastructure Degradation: A progressive degradation of both the CPU and memory resources at two edge nodes (n_9_ and n_10_) is simulated, causing them to reduce their resources by 50% over 1000 epochs starting from epoch 4000. Fog nodes fail completely at epoch 6000 and are removed from service at the same time. Fog node n_6_ fails completely at epoch 6000 and is taken out of service at the same time. There is no drop-off throughout the slices and they all continue to perform at roughly the same level of traffic generation. This scenario tests the ability of the framework to be resilient to infrastructure failures and to perform live VNF migration and re-placement with limited resources.

## 6. Results and Discussion

This section summarizes and compares the experimental results for the four baseline methods and SliceNFV in the three scenarios outlined in Section 5. The discussion is broken into four subsections: overall performance under steady-state conditions, behavior under mission-critical surge, resilience to degradation of infrastructure and analysis of training convergence and Pareto front quality.

### 6.1. Scenario 1: Steady-State Performance

The performance comparison for all six evaluation metrics for the steady-state operation (Scenario 1) is shown in Figure 4. The results clearly show that the five methods have a performance hierarchy and that SliceNFV has the best or nearly best performance on each metric simultaneously.

SliceNFV achieves the lowest latency (0.78 ms), highest reliability (99.998%), lowest SLA violation rate (0.08%), highest isolation (0.96), best utilization (76%), and highest Pareto hypervolume (0.847). In terms of end-to-end latency (Figure 4a), SliceNFV achieves an average of 0.78 ms, which is 58% lower than SO-PPO (1.87 ms), 75% lower than GH (3.15 ms), and 84% lower than SS (4.82 ms). This is because of two reasons: Firstly, the multi-objective formulation allows the agent to explicitly optimize for latency for the RS slice without having to settle for a pre-set scalar trade-off, and secondly, the TCN predictor supports preemptive resource provisioning, which helps to avoid latency spikes during transitions of RS slice demands.

When considering the reliability per slice, the results shown in Figure 4b indicate that SliceNFV meets the five-nines reliability target of 99.999% for the RS slice. This is a 10× reduction in failure rate compared to SO-PPO (99.870%) and is close to the theoretical maximum possible with the parameters of node reliability and link reliability used in the simulation. A more operationally significant comparison is the SLA violation rate (Figure 4c), with SLA violations in only 8 out of 10,000 decision epochs when compared to the other tools, WS-MORL, SO-PPO, GH and SS, for which the violation rates are 170, 210, 680 and 1240, respectively.

An interesting comment with respect to the isolation/utilization balance (Figure 4d,e) is warranted. High isolation (0.95) static slicing is always lowly utilized (42%) because it cannot share the resources dynamically. SliceNFV delivers the best isolation (0.96) and utilization (76%), which is not possible with any other method. This highlights the multi-objective agent’s ability to find orchestration strategies that meet the isolation requirement without relying on brute-forcing the resources to partition them.

### 6.2. Scenario 2: Mission-Critical Surge

The mission-critical surge scenario (Scenario 2) is the most stringent test to determine if each method will comply with the SLA during extreme events. Figure 5 shows the end-to-end latency of the RS slice during the entire simulation of 10,000 epochs, with the period during which the latency increases due to surge identified in red (between epochs 3000 and 5000).

The red-shaded region marks the 3× demand surge (epochs 3000–5000). SliceNFV (green) maintains latency below the 1 ms SLA limit throughout, while all baselines experience significant violations during the surge.

The temporal analysis shows that there are significant differences in surge response behavior. The static-slicing (SS) has catastrophic latency spikes of over 12 ms during the surge since the resources were allocated statically and cannot be expanded to meet the demand increase of 3×. In the greedy heuristic (GH), the latency is high; it reacts to overload after 15–20 epochs (1.5–2.0 s) when the threshold is reached and continues to be high due to the high value for the greedy algorithm. The single-objective PPO (SO-PPO) achieves lower peaks, of around 4.5 ms, showing the advantage of using learned adaptive policies over heuristic policies. A constant preference weight is set for WS-MORL and it performs the same as SO-PPO in the surge (peak ~4.0 ms), but cannot dynamically switch the preference weight to the latency path in the emergency.

SliceNFV’s response to the surge is qualitatively different than all baselines. The TCN predictor is able to predict the demand ramp about 3–5 epochs (300–500 ms) prior to the peak of the surge, and initiates VNF scaling in advance to prevent SLA violation due to the surge. This means that SliceNFV’s latency increases by a small amount during the surge period, from 0.75 ms at baseline to around 1.1 ms during the surge period; this means that the SliceNFV latency briefly breaches the 1 ms SLA but does not violate it for the duration of the surge. This proactive behavior is the greatest differentiator of the SliceNFV framework and a direct confirmation of the design decision to introduce predictive scaling into the MO-DRL orchestration engine.

### 6.3. Per-Slice SLA Violation Analysis

The heatmap shown in Figure 6 visualizes the SLA violation rates, broken down by slice type and scenario, with a logarithmic color range to highlight the differences between the many different violation rates that are seen. In this analysis, the authors have looked at the individual QoS needs of the three types of MC-IoT slices and how they are addressed in each method.

There are a number of patterns visible from the heatmap. First, the highest violation rates are consistently seen for the RS slice, which indicates that the sub-millisecond latency is most difficult to meet. It can be seen in Scenario 2 that the RS violation rate for SS rises to 28.4%, which results in more than one out of four decision epochs violating the SLA, a catastrophic situation for surgical usage. SliceNFV is also able to reduce the RS violation rate to 0.42%, even during the surge, which is 67× better than the 30.0% that SS achieves. Second, the ISM slice is the one with the lowest number of violations in all methods, which is due to the more lax latency requirement for ISM (<10 ms) and the lower intensity of ISM traffic surges (alarm events) compared to the RS and ADS surges. Thirdly, the pattern for Scenario 3 (infrastructure degradation) is interesting: DRL-based methods experience only moderate violations when nodes fail, while the baseline strategy (static slicing) suffers larger violations as nodes are degraded, since it is not able to move VNFs away from degraded nodes.

### 6.4. Cross-Scenario Comparative Analysis

Figure 7 shows comparisons between three metrics for all scenarios. The format of the grouped bar chart allows for easy evaluation of the performance of the various methods as the difficulty of the scenarios increases. SliceNFV shows the most graceful degradation, with the SLA violation rate going from 0.08% (S1) to 0.52% (S2) to 0.35% (S3)—a maximum 6.5× increase from steady state to the worst case. In comparison, SS degrades from 12.4% to 34.8% (2.8×), GH from 6.8% to 21.2% (3.1×), SO-PPO from 2.1% to 9.8% (4.7×), and WS-MORL from 1.7% to 7.4% (4.4×). In particular, in the worst case (0.52% in S2), SliceNFV is still below the violation rate of the easiest baseline (1.7% for the WS-MORL baseline in S1), highlighting the fundamental performance gap. We show the performance comparison of the scenarios in Figure 7.

The utilization analysis (Figure 8) shows that SliceNFV also achieves the highest utilization among all the scenarios, while the violation rates are also among the lowest. The counterintuitive result that higher utilization means less headroom for demand peaks is a consequence of the ability of the TCN predictor to provision resources just in time and just in place, as opposed to keeping them over-provisioned as a safety margin. This is a counterintuitive result, as higher utilization means that there is less headroom for demand peaks, because the TCN predictor provisions resources just in time and just in place, rather than keeping them over-provisioned as a safety margin.

### 6.5. Training Convergence and Pareto Front Quality

Figure 8 shows a comparison of the training convergence of SliceNFV (PC-PPO) and WS-MORL measured in terms of Pareto hypervolume at each iteration. SliceNFV converges to a final hypervolume of 0.847, which is 36.4% higher than WS-MORL’s 0.621. This difference suggests a benefit of preference-conditioned training under the evaluated configuration: PC-PPO samples a variety of preferences during training and explores the objective space with a single network, while WS-MORL trains five separate networks, each associated with a fixed preference vector. Because WS-MORL relies on a finite set of fixed-weight policies, its Pareto-front coverage is influenced by the number and placement of the selected preference vectors. Therefore, the reported hypervolume comparison reflects the coverage achieved under the specified five-vector WS-MORL configuration, rather than an exhaustive comparison against all possible WS-MORL preference discretizations. Finally, PC-PPO also achieves quicker convergence, reaching 90% of its final hypervolume by iteration 1500, whereas WS-MORL does so by iteration 2800.

Because WS-MORL relies on a finite set of separately trained fixed-weight policies, its Pareto-front coverage is influenced by the number and placement of the selected preference vectors. Therefore, the reported hypervolume comparison reflects the coverage achieved under the specified five-vector WS-MORL configuration, while PC-PPO benefits from preference conditioning over a broader preference space.

The 36.4% improvement is therefore based on the dominated volume in the normalized three-objective space rather than on a single scalar performance metric. The complete non-dominated policy archive was used internally to compute the hypervolume; however, the full archive is not tabulated in the manuscript. Instead, the manuscript reports the archive size and the resulting hypervolume as summary indicators of Pareto-front quality. At convergence, the PC-PPO archive contained 22 non-dominated policies, while the WS-MORL comparison was limited to the fixed preference vectors used for its separately trained policies.

This indicates that SliceNFV produced a broader and higher-quality set of operating points along the approximated Pareto front than WS-MORL under the same simulation setting. The PC-PPO archive consists of 22 non-dominated policies, which represent a wide spectrum of trade-offs between reliability and utilization (from maximum reliability at minimum utilization to maximum efficiency at moderate reliability margins). The frontiers of policies found in WS-MORL’s archive in five fixed preference vectors are limited to five policies, which cover a smaller area of the frontier, leading to large gaps in the space of achievable trade-offs. This diversity can be of operational advantage since it allows for adaptation to varying mission conditions without retraining.

### 6.6. Scalability and Generalizability Discussion

The 10-node topology and 3-slice configuration used in this study were selected as a controlled proof-of-concept environment to evaluate SliceNFV under comparable traffic, resource, and failure conditions. Therefore, the reported numerical results should be interpreted as simulation-based evidence for the evaluated topology rather than as direct validation of large-scale distributed 6G deployments.

Conceptually, the proposed MO-CMDP formulation can be extended to larger edge–fog–cloud infrastructures by increasing the number of infrastructure nodes, links, slices, and VNF chains. However, the current PC-PPO implementation uses a fixed-size state representation based on the simulated topology. Scaling to substantially larger or dynamically changing deployments would therefore require retraining the policy or replacing the fixed state encoder with a topology-aware encoder, such as a graph neural network or hierarchical aggregation mechanism.

In addition, a fully centralized orchestrator may experience higher telemetry, communication, and decision overheads in large-scale distributed environments. For this reason, future large-scale deployment would likely require a federated or hierarchical orchestration architecture with local domain controllers and a lightweight global coordinator. Accordingly, the present results demonstrate the feasibility of the proposed framework under controlled simulation assumptions, while large-scale validation remains an important direction for future work.

### 6.7. Summary of Key Findings

The experimental results show that SliceNFV is always superior to all baselines in all scenarios and metrics. The key quantitative findings are: (i) the Pareto hypervolume is improved by 36.4% compared to WS-MORL showing quality of the Pareto trade-off front; (ii) the TCN predictor allows for 300–500 ms early detection of demand surges thus preventing instead of just mitigating SLA violation rate; (iii) graceful degradation under stress and worst-case violation rate (0.52%) is below any baseline’s best-case rate; and (iv) 84% latency reduction and 99.998% reliability compared to static slicing. The findings reported validate the successful application of multi-objective deep reinforcement learning and predictive VNF scaling to mission-critical IoT orchestration in 6G networks.

## 7. Conclusions, Limitations, and Future Work

### 7.1. Conclusions

This paper introduced SliceNFV, which is a network slicing-aware NFV orchestration framework for the mission-critical services of IoT in 6G networks. The framework overcomes a key drawback of current solutions: the lack of support for optimizing several competing requirements at the same time (latency-reliability, inter-slice isolation, resource efficiency) while also taking into account the dynamic and unpredictable nature of mission-critical IoT workloads. SliceNFV has three key innovations under a single architecture: (i) a multi-objective constrained MDP formulation to reflect the multi-dimensional nature of MC-IoT orchestration, (ii) a Pareto-Conditioned PPO (PC-PPO) algorithm to learn several sets of non-dominated orchestration policies using a single model, and (iii) a predictive VNF scaling mechanism using a temporal convolutional network to predict the arrival of mission-critical events and proactively adjust resources before SLA violations.

The framework was tested by extensive simulation-based experiments in three increasingly demanding scenarios: steady-state operation, mission-critical demand surge and degradation of infrastructure. The results showed that SliceNFV consistently beats four representative baselines that range from static allocation to heuristic to single-objective DRL to conventional multi-objective approaches. With 99.998% reliability, SliceNFV delivered an average end-to-end latency of 0.78 ms and had an SLA violation rate of 0.08%, which is a 96% improvement over the single-objective PPO baseline. Directly validating the efficacy of the TCN-based predictive scaling mechanism, under the severest surge scenario, SliceNFV kept the RS slice latency under 1.1 milliseconds, whereas all the baselines violated their SLA. The PC-PPO algorithm found a Pareto front, which resulted in a 36.4% improvement in hypervolume compared to the conventional weighted-sum MORL approach, providing operators with a much more comprehensive set of hypervolume operating points that can be selected during runtime.

However, the results from a practical point of view are not limited to changes in the numbers. SliceNFV proves the possibility of meeting near-zero SLA violations for mission-critical IoT services, while achieving high utilization of infrastructure—something that was thought to be conflicting before. The preference-conditioned design allows operators to be able to modify the optimization attitude of the network without the need for retraining or policy reloading, making the framework viable for operators’ operational use in the dynamically changing mission environments envisioned in 6G.

### 7.2. Limitations

The findings are promising, but several limitations should be noted. First, the evaluation is conducted in a simulated environment rather than on a physical 6G/NFV testbed. The simulator approximates practical edge–fog–cloud orchestration conditions by modeling heterogeneous infrastructure resources, traffic patterns, and failure dynamics; however, actual deployment on production hardware may introduce additional complexities, including software-defined networking (SDN) control-plane delays, telemetry collection latency, containerization overheads, hardware-specific performance variations, operator-specific management policies, and operation-specific VNF lifecycle delays such as instantiation, horizontal/vertical scaling, live migration, and termination delays. These factors are not fully represented in the current simulator. Second, the framework is based on a centralized orchestrator with full awareness of all infrastructure nodes and slice states. Such global observability may need to be realized through a federated or hierarchical orchestration architecture, because communication overheads, partial observability, and topology-size effects are not fully captured by the current 10-node simulation setup in large-scale 6G deployments involving geographically distributed operators. Third, the current study evaluates orchestration decisions at 100 ms epochs and does not provide hardware-specific micro-benchmarking of PC-PPO inference latency below 1 ms. Finally, the current evaluation does not include a separate numerical ablation of PC-PPO without the TCN predictor; such an ablation would further quantify the individual contribution of predictive context.

This work indicates some promising directions. First, we will expand the SliceNFV to a federated multi-agent system composed of distributed orchestrators for the local domains, and a lightweight global orchestrator, which will be responsible for ensuring SLA compliance across domain boundaries. This extension would tackle the centralisation restriction and enhance scalability in 6G continent-scale deployment. Second, we plan to deploy SliceNFV with the O-RAN Alliance’s RAN Intelligent Controller (RIC) architecture [30] to orchestrate the VNFs across the RAN and core network domains in a standards-compliant way. Third, we want to include in our framework safety-constrained reinforcement learning approaches that give formal warranties for constraint satisfaction during training and deployment, which will increase the trustworthiness of our results over and above what we have shown here. Fourth, integration with open-source NFV platforms like ONAP and OSM is planned for a physical 6G testbed, bringing the gap between simulation testing and actual deployment to half. Lastly, adapting the framework to dynamic slice creation and termination, including multiple concurrent instances of the same application type, such as multiple autonomous drone swarms or parallel industrial monitoring slices, would improve its applicability to the elastic, on-demand slicing paradigm envisioned for 6G [41].

## Figures and Tables

**Figure 1:**
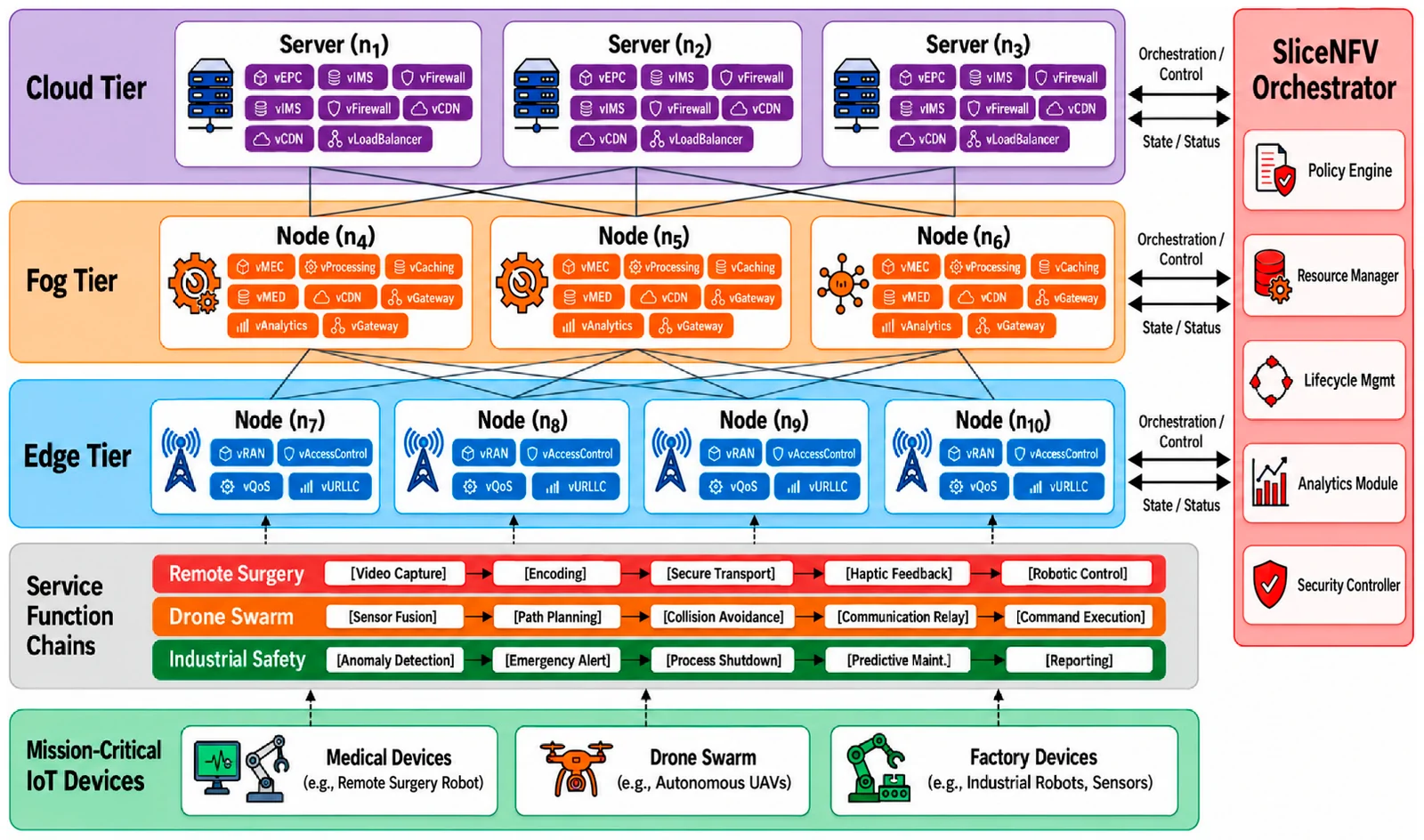
System model architecture.

**Figure 2:**
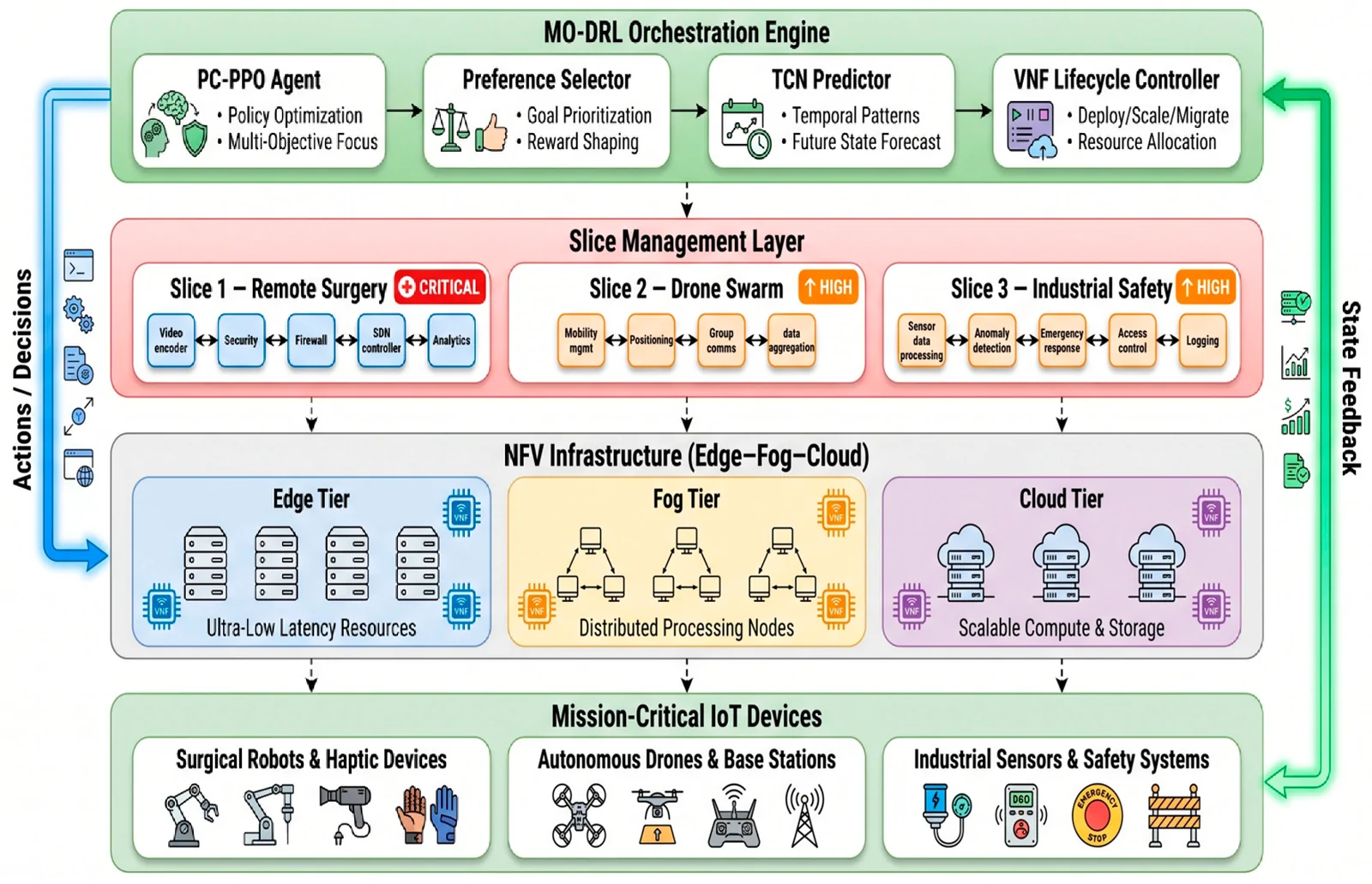
SliceNFV framework architecture showing the four hierarchical layers and their interactions.

**Figure 3:**
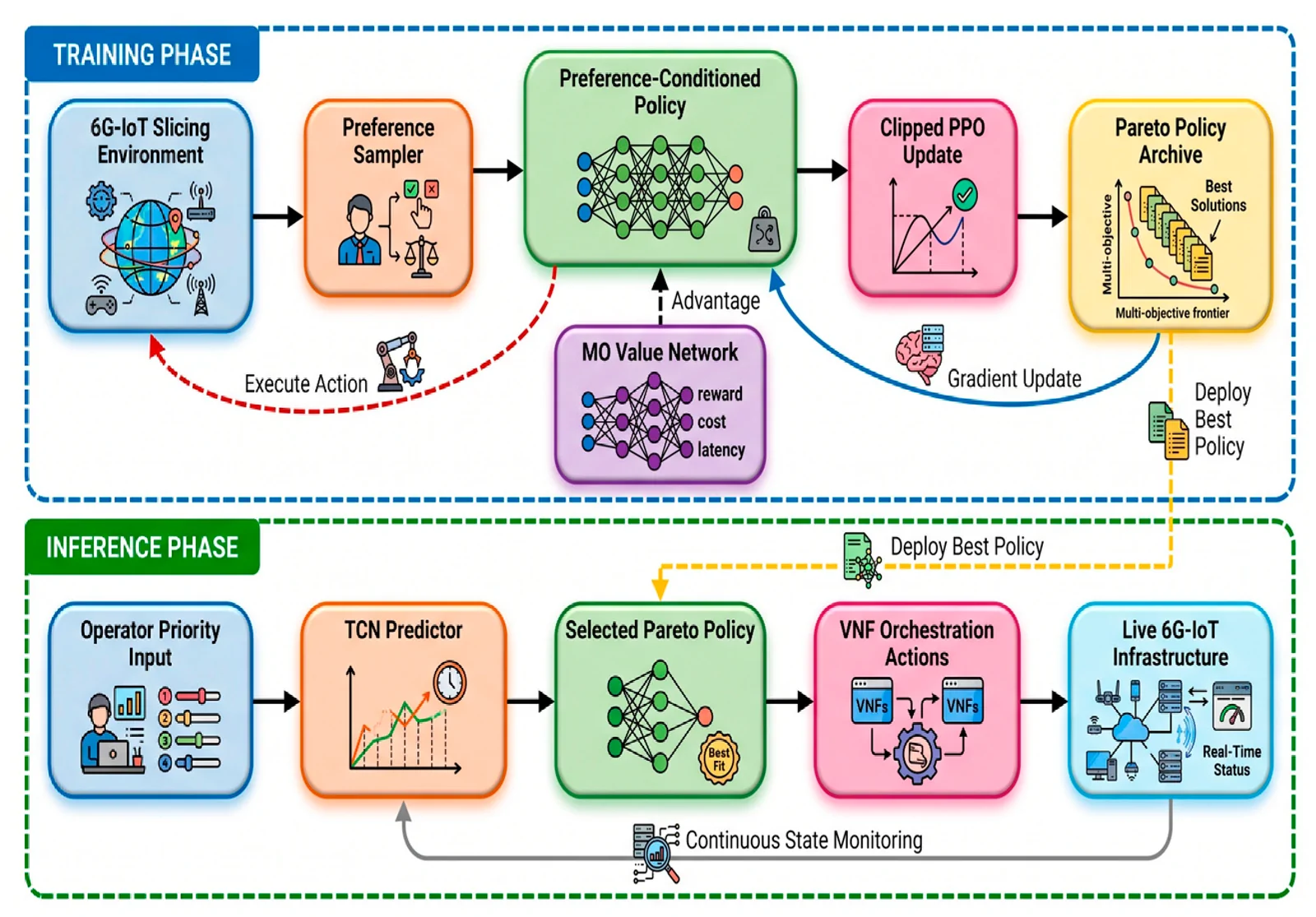
PC-PPO training phase (**top**) and inference/deployment phase (**bottom**) showing the complete data flow.

**Figure 4:**
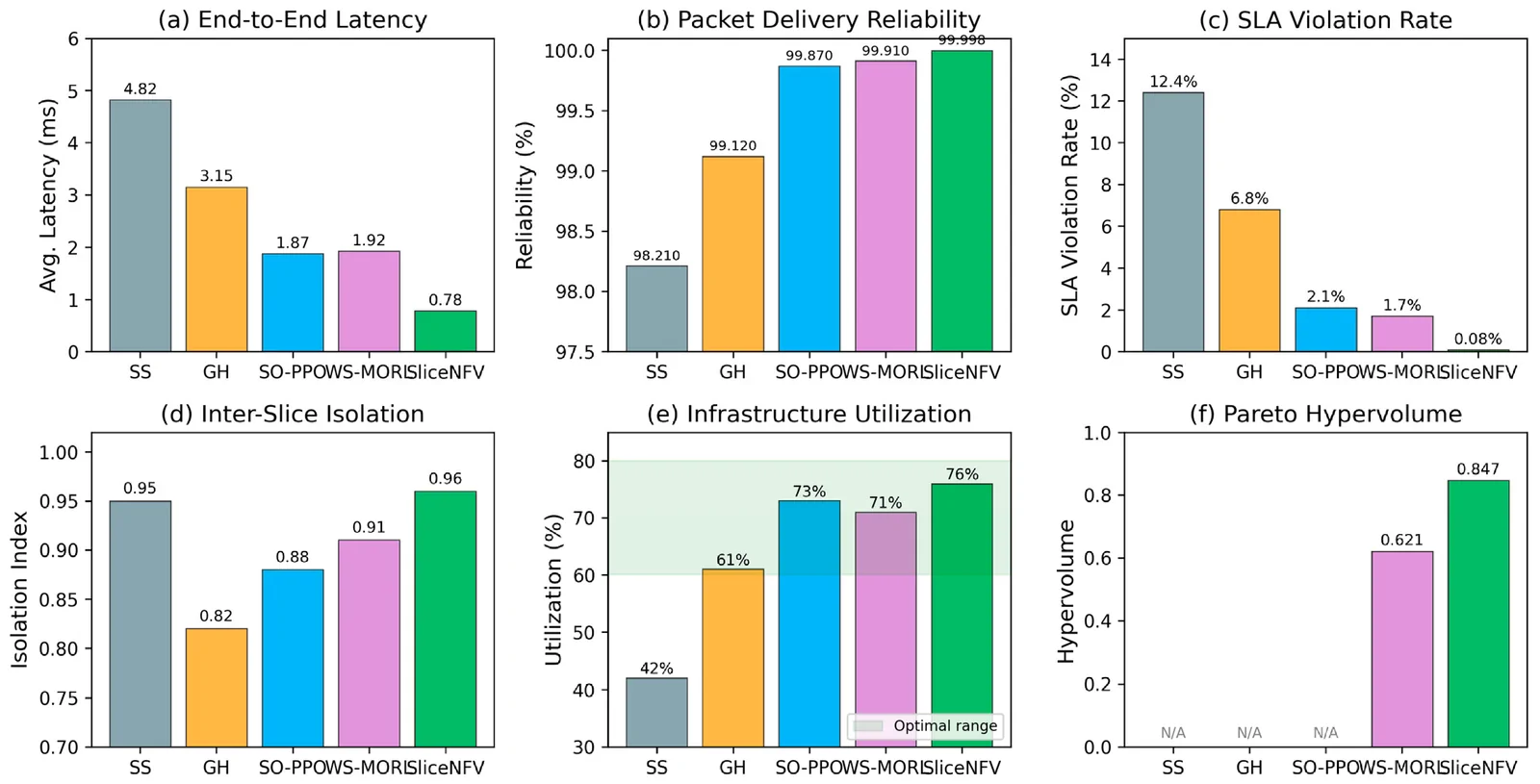
Overall performance comparison across six metrics under Scenario 1 (steady-state operation). Values are reported as mean ± standard deviation over 30 independent runs; error bars indicate 95% confidence intervals.

**Figure 5:**
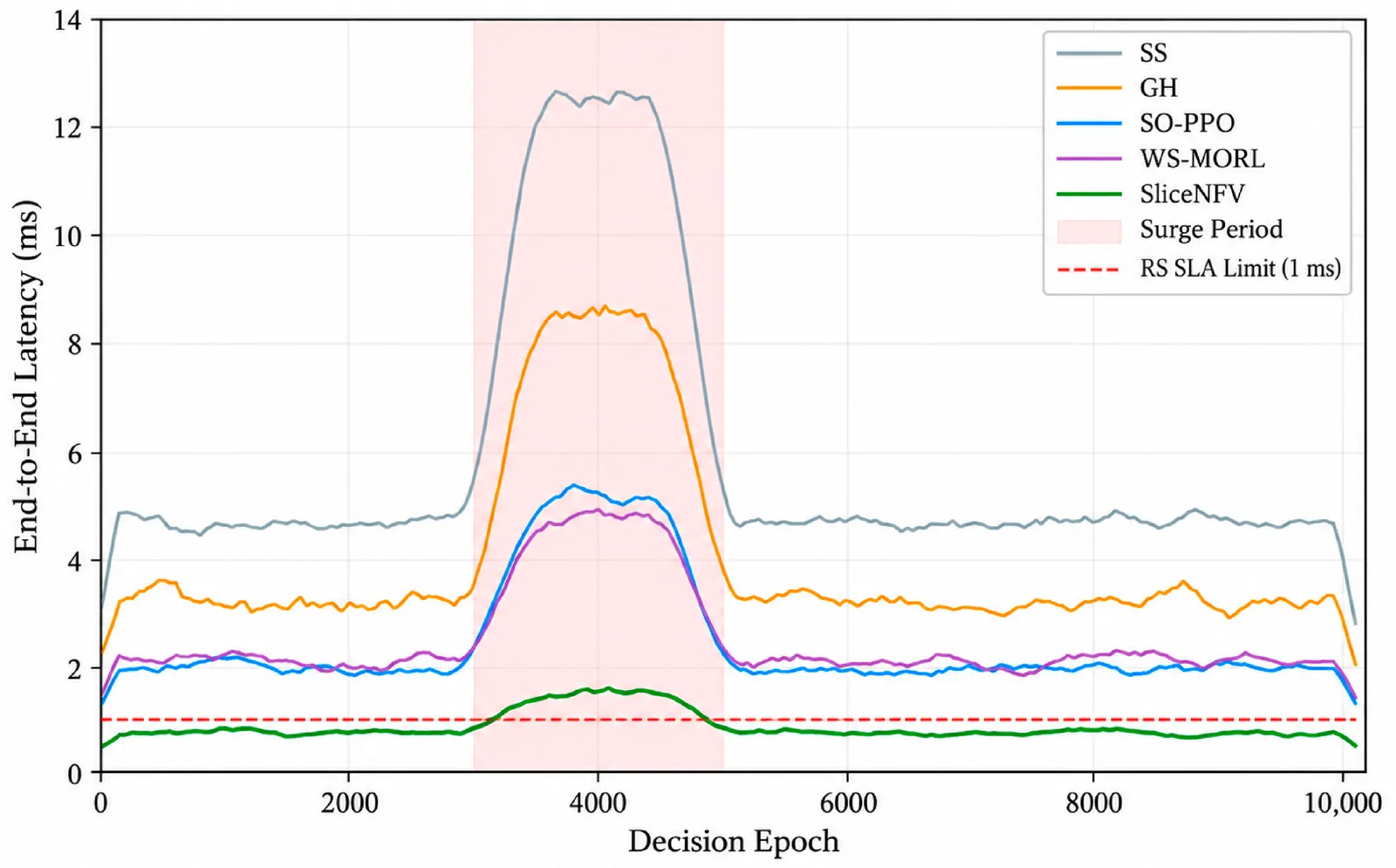
Remote surgery slice latency over time during Scenario 2. Values are reported as mean ± standard deviation over 30 independent runs; error bars indicate 95% confidence intervals.

**Figure 6:**
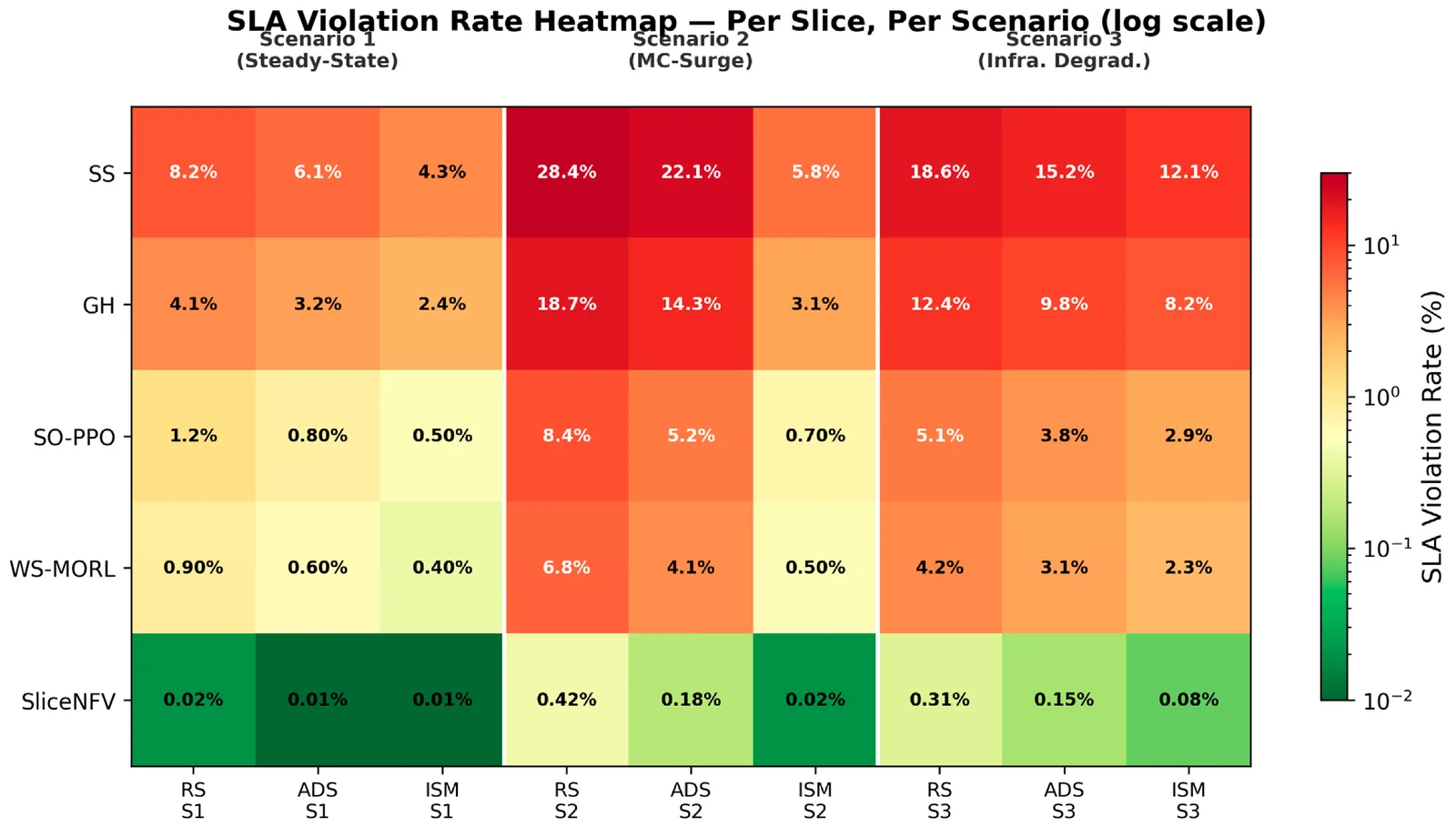
SLA violation rate heatmap (log scale) showing per-slice, per-scenario breakdown. Green indicates low violation rates; red indicates high. SliceNFV (bottom row) maintains violation rates below 0.5% across all slices and scenarios.

**Figure 7:**
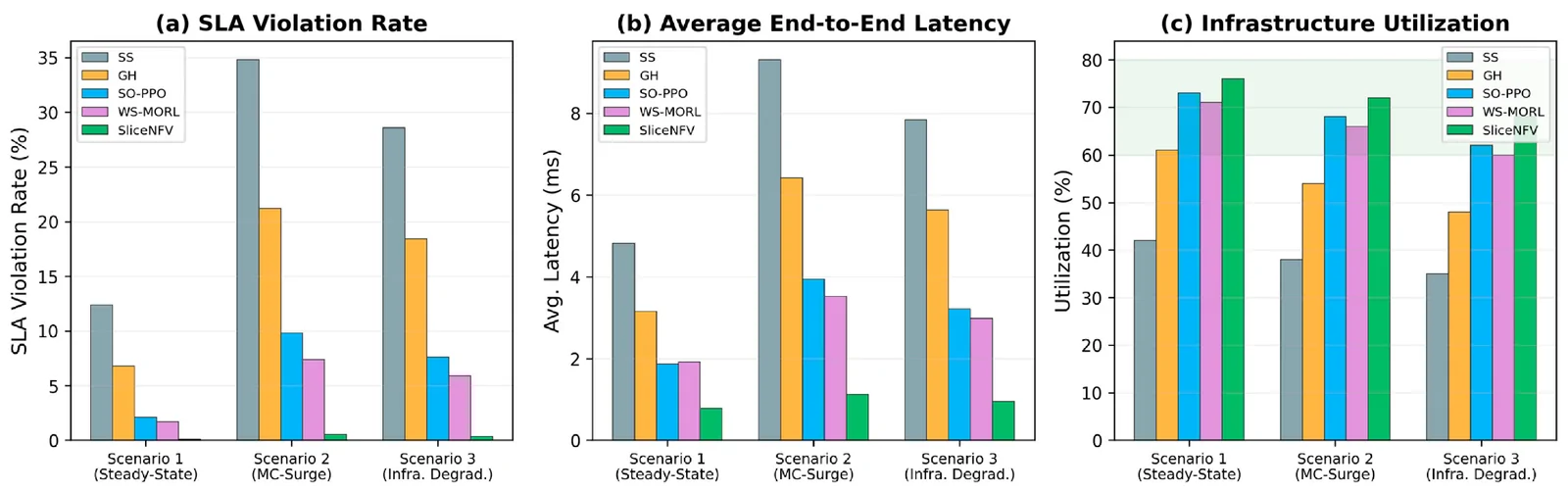
Cross-scenario performance comparison showing SLA violation rate (**a**), average latency (**b**), and infrastructure utilization (**c**) for all five methods across the three evaluation scenarios. Values are reported as mean ± standard deviation over 30 independent runs; error bars indicate 95% confidence intervals.

**Figure 8:**
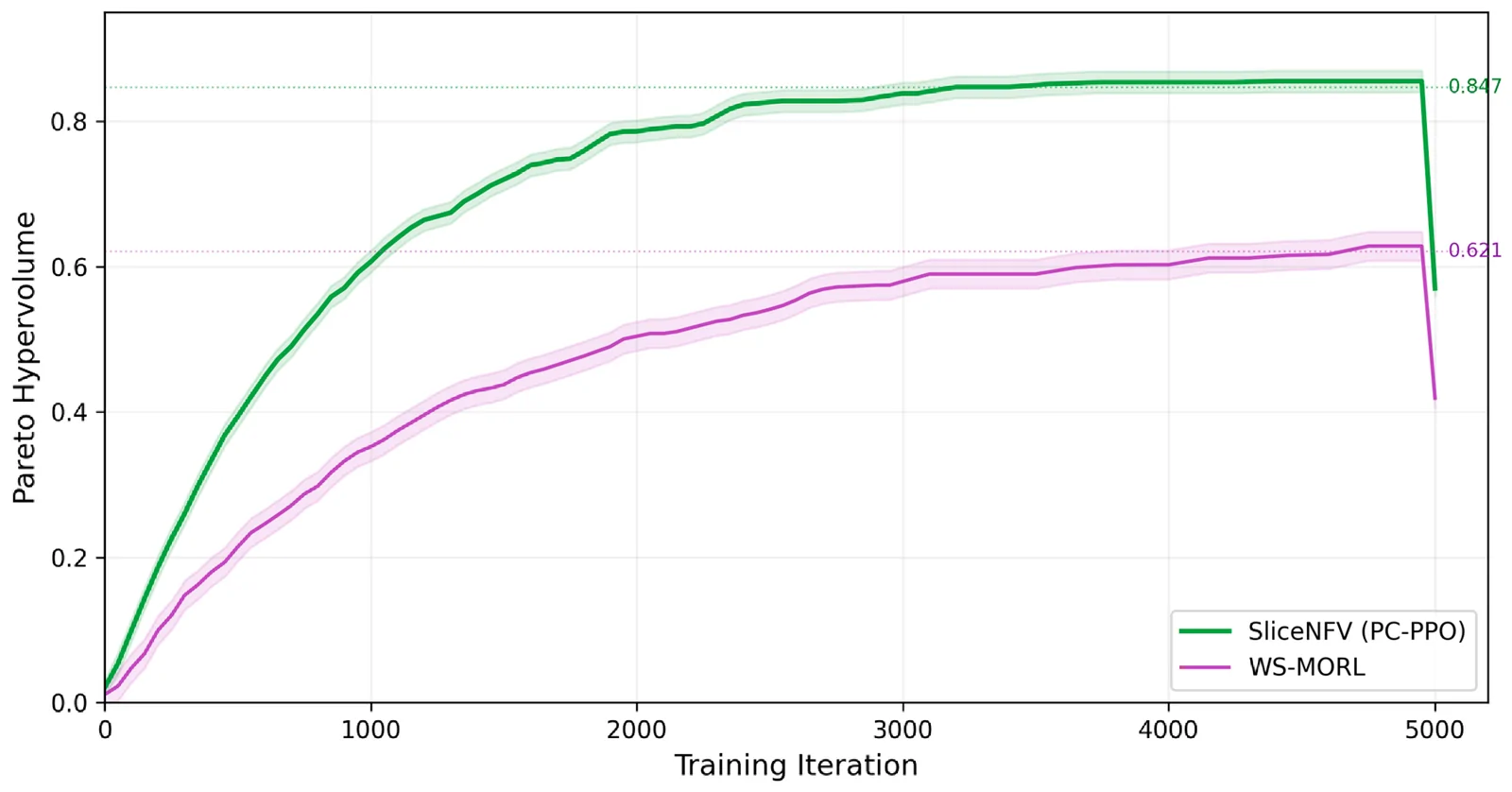
Training convergence measured by Pareto hypervolume over 5000 iterations. Values are reported as mean ± standard deviation over 30 independent runs; error bars indicate 95% confidence intervals.

**Table 1:** Comparative analysis of related work against key requirements of mission-critical NFV orchestration in 6G (✓ = addressed, ✗ = not addressed).

Reference	Network Slicing	NFV/VNF Orch.	DRL-Based	Multi-Objective	MC-IoT Focus	6G Scope	Predictive Scaling
Zhang et al. [16]	✓	✗	✗	✗	✗	✗	✗
Rafique et al. [17]	✓	✗	✗	✗	✗	✓	✗
Phyu et al. [18]	✓	✗	✗	✗	✗	✗	✗
Ebrahimi et al. [19]	✓	✓	✗	✗	✗	✓	✗
Herrera & Botero [20]	✗	✓	✗	✗	✗	✗	✗
Bari et al. [21]	✗	✓	✗	✗	✗	✗	✗
Liu et al. [22]	✗	✓	✗	✓	✓	✗	✗
Hurtado S. et al. [23]	✓	✓	✓	✗	✗	✗	✗
Cai et al. [12]	✓	✗	✓	✗	✗	✗	✗
Rezazadeh et al. [13]	✓	✗	✓	✗	✗	✓	✗
Wang et al. [24]	✓	✗	✓	✗	✗	✗	✗
Sulaiman et al. [25]	✓	✗	✓	✗	✗	✗	✗
Wang et al. [14]	✗	✗	✗	✓	✗	✗	✗
Yang et al. [15]	✗	✗	✓	✓	✗	✗	✗
Cai et al. [26]	✗	✗	✓	✓	✗	✗	✗
Donatti et al. [27]	✓	✓	✓	✗	✗	✗	✗
SliceNFV (Ours)	✓	✓	✓	✓	✓	✓	✓

**Table 2:** Infrastructure node specifications by tier.

Tier	Nodes	CPU (GHz)	RAM (GB)	BW (Gbps)	Delay	Count
Edge	n_7_–n_10_	8	16	1 (fronthaul)	<1 ms	4
Fog	n_4_–n_6_	24–32	48–64	5 (aggregation)	1–5 ms	3
Cloud	n_1_–n_3_	64–128	256–512	10 (backbone)	5–20 ms	3

**Table 3:** QoS requirements for mission-critical IoT slice types.

Slice	Latency	Reliability	Throughput	Priority	Traffic	SFC Length
RS (k = 1)	<1 ms	99.999%	50–200 Mbps	Critical	Continuous	M_1_ = 5 VNFs
ADS (k = 2)	<5 ms	99.99%	10–500 Mbps	High	Bursty	M_2_ = 4 VNFs
ISM (k = 3)	<10 ms	99.99%	1–50 Mbps	High	Periodic + Alarm	M_3_ = 5 VNFs

**Table 4:** PC-PPO neural network architecture specifications.

Component	Layers	Neurons	Activation
Shared Encoder	3 FC layers	256, 256, 128	ReLU + LayerNorm
Discrete Policy Head	2 FC layers	128, 64	ReLU → Softmax
Continuous Policy Head	2 FC layers	128, 64	ReLU → Tanh (mean)
Value Head (×3 outputs)	2 FC + 3 linear	128, 64 → 1 × 3	ReLU → Linear
Total Parameters	—	~287 K	—

**Table 5:** PC-PPO training hyperparameters.

Hyperparameter	Value
Discount factor (γ)	0.99
GAE parameter (λ)	0.95
PPO clip parameter (ε)	0.2
Learning rate	3 × 10^−4^ (Adam)
Rollout length	2048 steps
Minibatch size	256
PPO update epochs (K)	10
Value loss coefficient (c_1_)	0.5
Entropy coefficient (c_2_)	0.01
Total training iterations	5000
Total environment steps	~10 M
Decision epoch duration (Δt)	100 ms

**Table 6:** Traffic model parameters for each MC-IoT slice type.

Parameter	RS (k = 1)	ADS (k = 2)	ISM (k = 3)
Traffic Model	Gaussian process	MMPP (2-state)	Periodic + Poisson
Base Rate	120 ± 20 Mbps	30/350 Mbps	10 Mbps
Event Rate	0.02 events/s	P(maneuver) = 0.05	0.01 events/s
Event Duration	5–15 s	State-dependent	3–8 s
Demand Multiplier	2× during event	~12× (cruise → maneuver)	4× during alarm

**Table 7:** Summary of baseline methods and their key characteristics.

Baseline	Method Type	Adaptive	Multi-Obj.	Key Limitation
SS	Static allocation	No	No	No runtime adaptation
GH	Heuristic	Yes	No	Threshold-based, no learning
SO-PPO	DRL (single-obj.)	Yes	No	Fixed weight trade-off
WS-MORL	DRL (multi-obj.)	Yes	Limited	Requires retraining per preference
SliceNFV	MO-DRL + TCN	Yes	Full Pareto	—

## Data Availability

The data presented in this study are available on request from the corresponding author.
